# Bias-programmable linear-to-nonlinear photoresponse for filter-free single-pixel VIS–NIR discrimination

**DOI:** 10.1186/s40580-026-00585-7

**Published:** 2026-10-26

**Authors:** Jiyoon Shin, Masroor Alam, Jaeyeon Kim, Yong Geon Lee, Min Kyu Jeon, Hyeonghun Kim, Gun Young Jung

**Affiliations:** 1 Department of Materials Science and Engineering (DMSE), Gwangju Institute of Science and Technology (GIST), Gwangju, 61005 Republic of Korea; 2 Center for Frontier AI Research (CFAR), Agency for Science, Technology and Research (A*STAR), Singapore, 138632 Singapore; 3 School of Chemical Engineering (SCE), Chonnam National University (CNU), Gwangju, 61186 Republic of Korea

**Keywords:** Dual-band photodiode, Spectral discrimination, Nonlinear photoresponse, Filter-free multispectral sensing, Bias-programmable photodetection, Interfacial current-path engineering, Perovskite, Perovskite–silicon photodetector, Nano-optoelectronics

## Abstract

**Abstract:**

Filter-free discrimination of visible (VIS) and near-infrared (NIR) light within a single photodetector pixel remains challenging because conventional photodiodes typically require optical filters or multiple detector elements. This work reports a monolithic nonlinear back-to-back photodiode (NL-BPD) that enables bias-programmable reconfiguration between high-linear-dynamic-range (LDR) photodetection and nonlinear spectral-discrimination modes. By changing the bias from − 0.5 V to + 0.5 V, the device's initially independent linear responses in both VIS and NIR channels (LDRs ≥ 100 dB) evolve into a nonlinear regime in which the VIS response reverses polarity with increasing light intensity while the NIR response remains monotonic. This behavior is enabled by defect-assisted transport pathways formed through partial interfacial reactions between the highly conductive PEDOT:PSS interlayer and MAPbI_3_. Together with the optimized device architecture, this interfacial current-path engineering reduces resistive bottlenecks and promotes cooperative carrier extraction from both absorbers. The resulting electrical signatures allow mixed VIS–NIR signals to be decoded from a single electrical output without optical filters or spatially separated pixels. The NL-BPD enables accurate spectral reconstruction (average R^2^ = 0.9996), demonstrates single-point pulse oximetry, and achieves > 96% accuracy in normalized-difference-vegetation-index-based vegetation health classification. These results establish bias-programmable nonlinear photodetection as an effective strategy for single-pixel multispectral sensing.

**Graphical abstract:**

**Figure:**
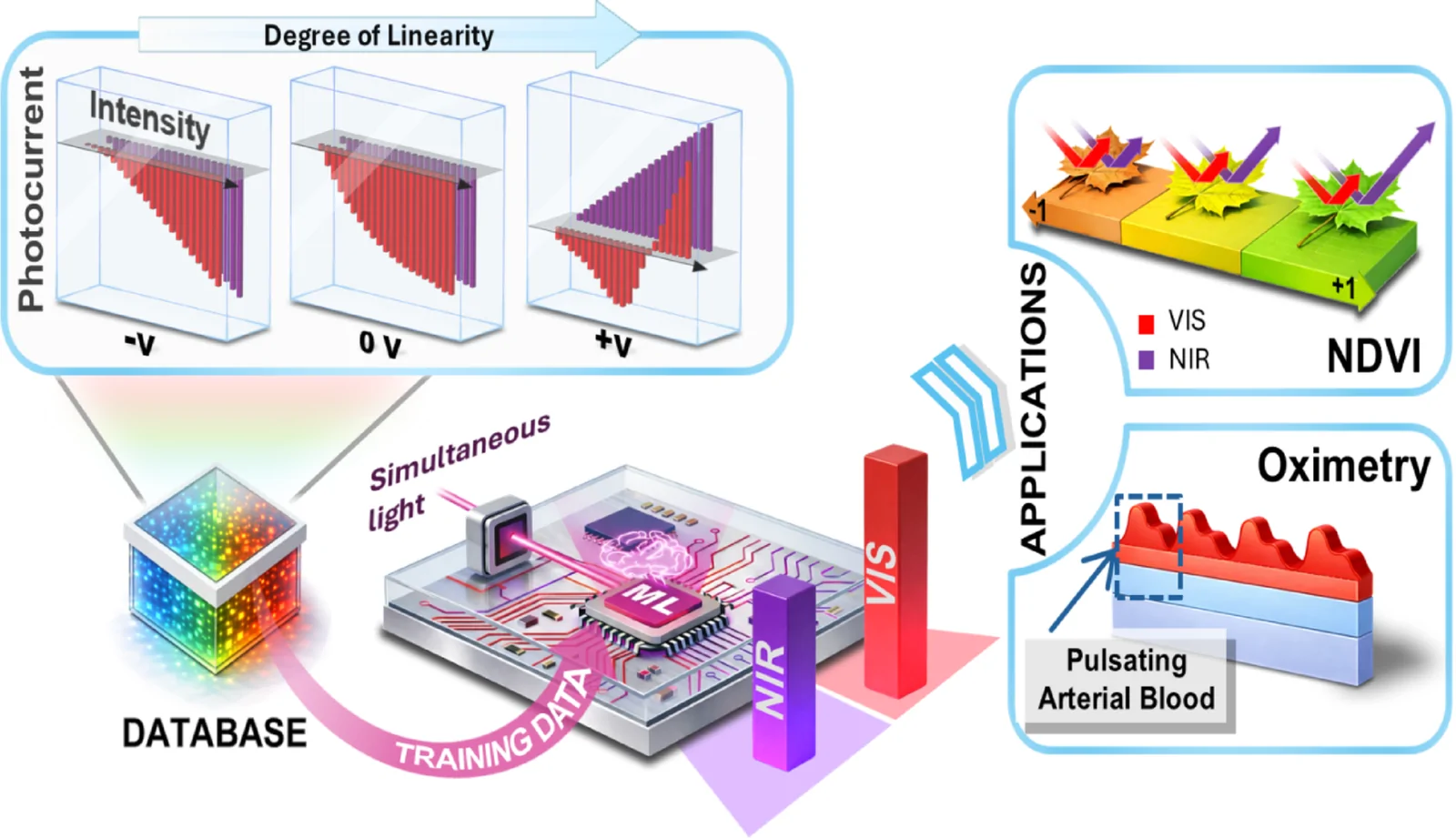

**Supplementary Information:**

The online version contains supplementary material available at https://doi.org/10.1186/s40580-026-00585-7.

## Introduction

Optical techniques provide an effective means for differentiating material properties through wavelength-dependent interactions between light and matter [1–3]. Access to such spectral information requires photodetectors capable of selectively resolving optical signals across different wavelength ranges [4–6]. If different wavelength components of incident light could be simultaneously discriminated and electrically separated within a single pixel, an integrated multifunctional optical sensing platform could be realized. Such a platform would enable diverse optical sensing tasks, including biomedical monitoring, agricultural diagnostics, environmental inspection, and machine-vision-based material identification, using a single compact device, without relying on complex optical architectures or multiple detector elements [6–12].

However, conventional photodetector technologies face fundamental challenges in separating and detecting wavelength-specific signals within a single device [11–13]. These limitations mainly originate from the broadband absorption characteristics of silicon (Si), which is widely used as an active photodetecting material [14–16]. Because Si photodetectors exhibit intrinsically weak wavelength selectivity, spectral information in most commercial systems is obtained through the integration of external optical filters. Therefore, filter-based approaches introduce optical losses, increase system complexity and power consumption, and impose structural constraints such as sensitivity to the angle of incidence [15–19].

To overcome these limitations, filter-free photodetectors that achieve wavelength selectivity through intrinsic material absorption or structural engineering have attracted considerable attention [20–27]. Within these approaches, bias-switchable dual-band photodetectors have emerged as promising platforms for compact spectral sensing because they enable wavelength-selective operation within a single device by simply changing the applied bias [26–30]. These devices typically incorporate two photoabsorbing layers with different bandgaps, allowing selective collection of photoresponses under opposite bias polarities. Among them, monolithic back-to-back photodiodes (BPDs) featuring vertically stacked junctions with opposing polarities (e.g., PNIP or PINIP) and bias-switchable organic photodetectors (OPDs) based on stacked visible (VIS)- and near-infrared (NIR)-absorbing layers are widely investigated architectures for realizing bias-switchable dual-band operation [31, 32].

However, the majority of vertically stacked bias-switchable dual-band photodetectors are inherently constrained by their serial junction configuration. Under a given bias polarity, only one junction is preferentially activated for charge collection, while the oppositely biased junction behaves as a resistive element [33]. This large series resistance impedes carrier extraction, reduces responsivity, weakens spectral signal separation, and limits the linear dynamic range (LDR) [34, 35]. Consequently, relatively high operating voltages (> 1 V) are typically used to sustain measurable photocurrents while maintaining a broad LDR (typically 80–120 dB), which generally increases dark current and noise, creating a trade-off between operating voltage, efficiency, and responsivity [36–40]. Moreover, the confinement of their photoresponse to a single current-generation pathway limits the diversity of wavelength-specific electrical fingerprints that can be generated within a single pixel.

**Fig. 1:**
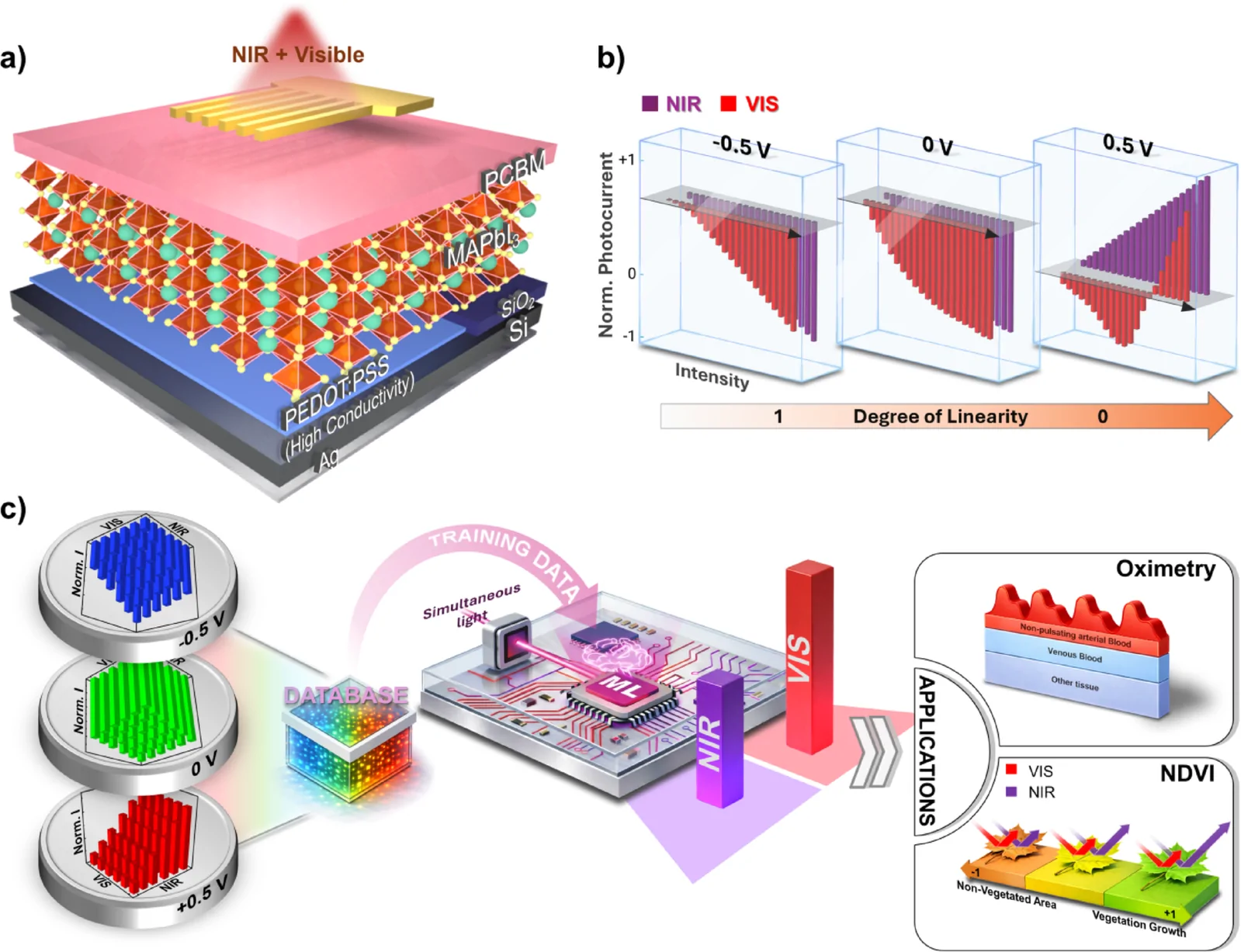
Device architecture and sensing concept of the bias-programmable NL-BPD. **a** Schematic of the monolithic perovskite-silicon hybrid photodiode featuring an NP^+^IN configuration. **b** Illustration of the normalized photoresponse characteristics of the photodiode under varying light intensities and low multi-bias conditions (− 0.5 V, 0 V, +0.5 V). **c** Schematic workflow showing the transition from raw data acquisition to system-level applications. A comprehensive spectral database is established across three distinct operating biases and subsequently used to train machine-learning algorithms for high-fidelity wavelength decoupling, enabling sensing applications such as oximetry and NDVI

Herein, we propose a monolithic nonlinear back-to-back photodiode (NL-BPD) based on a structurally modified NP^+^IN architecture, designed to operate as a bias-programmable multispectral sensing platform whose photoresponse can be switched from high-linearity photodetection to nonlinear spectral discrimination depending on the applied bias. The core design principle is the introduction of a high-conductivity poly(3,4-ethylenedioxythiophene):poly(styrenesulfonate) (PEDOT:PSS) interlayer between the Si substrate and the optimized thin organic–inorganic perovskite (MAPbI_3_) layer (Fig. 1a). Partial interfacial reactions between semi-metallic PEDOT:PSS and the perovskite layer create additional current pathways near the interface and upper perovskite [41–47], resulting in photoactivation of both the perovskite and Si absorbers with facile charge transport from the Si junction to the top electrode [48]. Although such interface interactions and the inherent high leakage current of semi-metallic PEDOT:PSS are usually considered detrimental in conventional optoelectronics [41–46], herein they are deliberately exploited to overcome the resistive bottleneck of conventional BPDs and enable carrier extraction from both absorption layers at low bias. As a result, the device exhibits electrically reconfigurable photoresponse characteristics governed by both wavelength and bias polarity. At −0.5 V, both VIS and NIR illumination produce linear photoresponse with LDRs ≥ 100 dB, whereas at +0.5 V, the VIS response becomes strongly nonlinear and exhibits polarity reversal while the NIR response remains monotonic.

Leveraging this bias-dependent transition between high-linearity photodetection and nonlinear responsivity fingerprinting, we establish a filter-free sensing platform that selectively decouples mixed VIS–NIR signals using their distinct electrical signatures (Fig. 1b). As a proof of concept, we demonstrate a single-source, single-detector multifunctional sensing system for pulse oximetry and normalized difference vegetation index (NDVI) monitoring, which would otherwise require complex multi-component optical systems (Fig. 1c; Figure S1, Supporting Information) [49–52]. These results establish the NL-BPD as a compact, low-power, and high-performance optoelectronic device for integrated multifunctional sensing.

## Results and discussion

### Monolithic NP^+^IN back-to-back photodiode architecture

**Fig. 2:**
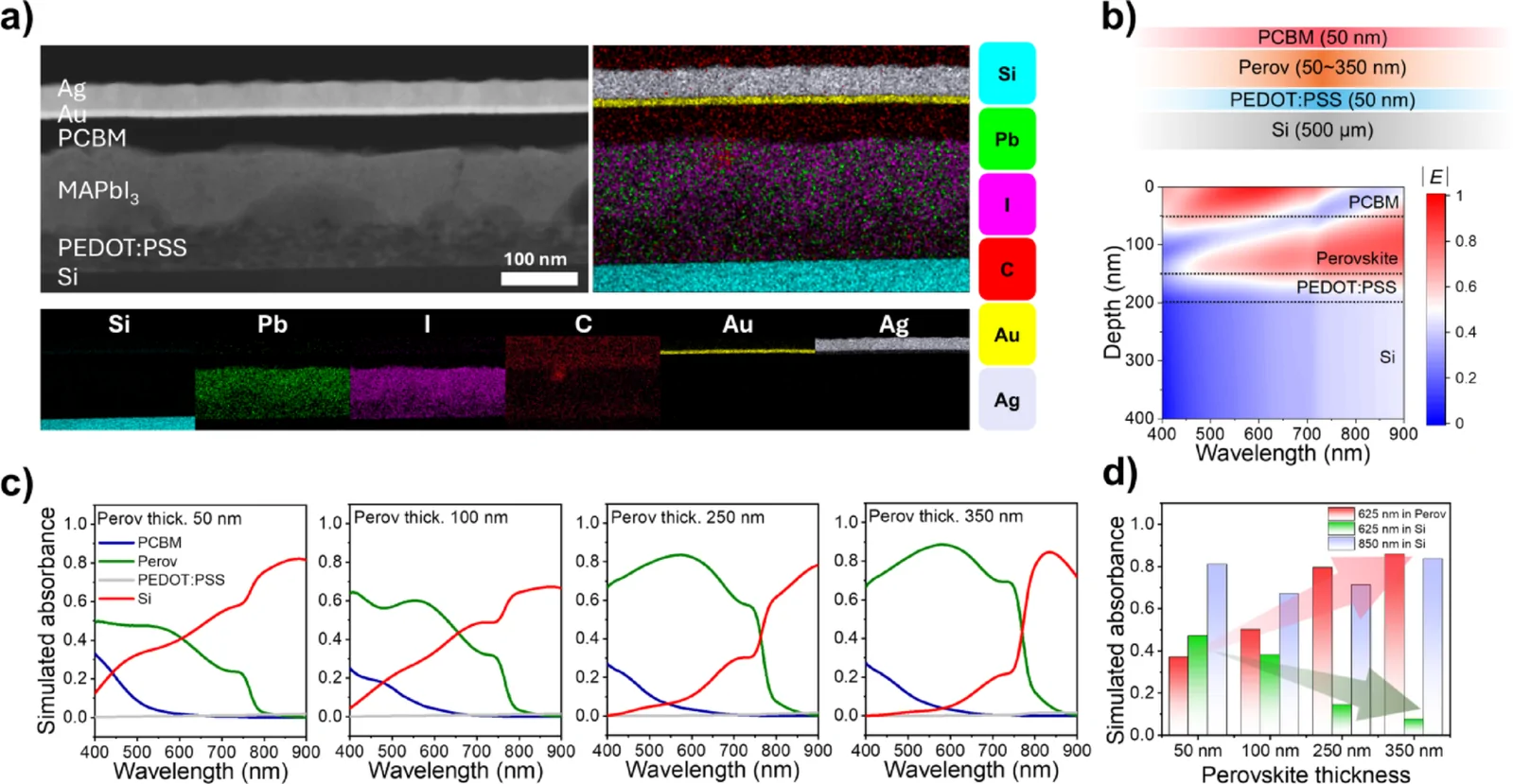
Structural characterization and optical design simulations. **a** HAADF-STEM image of the photodiode with corresponding EDS elemental mapping images for Si, Pb, I, C, Au, and Ag. **b** Simulated optical field (|*E*|) distribution within the full device architecture under monochromatic illumination from 400 to 900 nm. **c** Simulated layer-resolved optical absorption spectra for MAPbI_3_ thicknesses of 50, 100, 250, and 350 nm. **d** Summary of simulated absorbance at 625 nm and 850 nm for varying perovskite thicknesses

To achieve bias-programmable wavelength selectivity within a single monolithic stack, a vertical heterostructure was implemented in an NP^+^IN configuration by integrating high-conductivity PEDOT:PSS (HCP) as a low-resistivity interlayer between the n-Si substrate and the MAPbI_3_ perovskite absorber. The functional layers were deposited via solution processing, providing simple and scalable route for cost-effective large-area device integration [53]. Cross-sectional scanning transmission electron microscopy (STEM) using high-angle annular dark-field (HAADF) imaging reveals a well-defined layered architecture consisting of Ag/Au top electrodes (≈60 nm), PCBM (≈50 nm), MAPbI_3_ perovskite (≈100 nm), and PEDOT:PSS (≈50 nm) on the Si substrate (≈500 μm) (Fig. 2a). Energy-dispersive X-ray spectroscopy (EDS) elemental mapping confirms that Pb and I are predominantly localized in the MAPbI_3_ absorber layer, and the overall elemental distribution is consistent with the nominal layer sequence. The formation of a well-crystallized MAPbI_3_ layer was confirmed by X-ray diffraction (XRD) (Figure S2, Supporting Information) [54].

Transfer matrix simulations were performed under monochromatic illumination (400–900 nm) to map the optical field distribution within the NP^+^IN stack, thereby providing a physical basis for the observed wavelength selectivity (Fig. 2b–d) [55]. The complex refractive indices (n, k) of each layer (MAPbI_3_, PCBM, and Si [38]; PEDOT:PSS [56]; and Au [57]) were obtained from the literature. The simulated normalized field magnitude (|*E*|) as a function of wavelength and depth reveals that visible light is largely confined to the upper perovskite/PCBM region. With increasing wavelength toward the NIR region, the optical field penetrates deeper into the Si layer, indicating enhanced optical absorption in silicon at longer wavelengths. This wavelength-dependent field penetration provides the optical basis for the VIS/NIR-selective electrical response. The simulated absorption profile is consistent with the optical bandgap of MAPbI_3_ (≈1.59 eV; Figure S3, Supporting Information), which limits its absorption in the NIR region.

The absorption profiles of perovskite and silicon determine where the photo-generated carriers are generated and how they are subsequently transported. To investigate these absorption dynamics, layer-resolved simulations were conducted for perovskite thicknesses ranging from 50 to 350 nm (Fig. 2c, d), while keeping the thicknesses of other layers fixed. As the perovskite thickness increases, absorption at 625 nm is increasingly localized within the MAPbI_3_ layer, minimizing the amount of unabsorbed visible light transmitted to the underlying silicon. Meanwhile, absorption at 850 nm follows the trend consistent with Fig. 2b. To achieve bias-dependent photoresponses, as observed in previously reported BPDs [26, 28, 31, 36, 40], balanced carrier generation from both the perovskite and silicon layers is required. The perovskite layer must absorb sufficient visible light to contribute effectively to the visible photoresponse, while still allowing part of the incident light to reach the underlying silicon so that both layers can be photoactivated simultaneously. Simulation results suggest that a 100 nm MAPbI_3_ layer ensures this simultaneous excitation of both layers under visible light, which is essential for the bias-tunable photoresponse.

### Bias-dependent photodetection characteristics and device operation

To realize a low-voltage, bias-tunable photodiode architecture, it is essential to alleviate the resistive bottlenecks typically induced by the low-conductivity interlayers found in conventional vertically stacked photodiodes. In PEDOT:PSS, the PEDOT backbone provides electrical conductivity for charge transport, while insulating PSS serves as a counter-ion and dispersion stabilizer [58]. Thus, the charge-transport capability of the interlayer is fundamentally governed by the PEDOT-to-PSS weight ratio [59]. High-conductivity PEDOT:PSS (HCP, PH1000, PEDOT:PSS = 1:2.5 by weight) has traditionally been used as a transparent electrode in optoelectronic devices [60–62]. In contrast, previously reported BPD structures typically utilize a standard low-conductivity form (LCP, AI4083, PEDOT:PSS = 1:6 by weight) as the hole-transport layer [23, 26, 63]. To clarify the effect of interlayer conductivity in stacked photodiode architectures, devices incorporating HCP and LCP interlayers were directly compared.

**Fig. 3:**
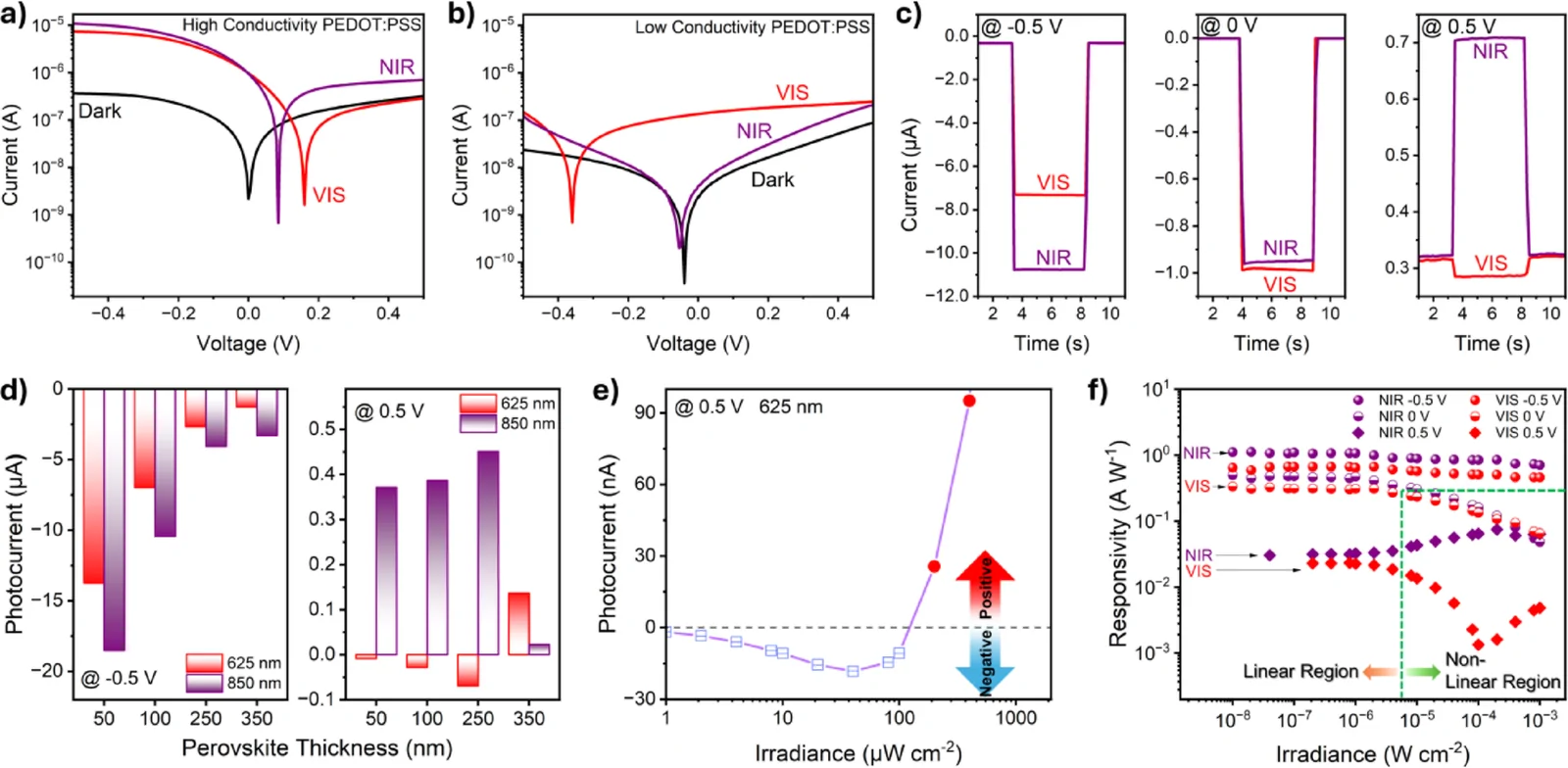
Bias-dependent photodetection performance and device optimization. **a**, **b** *I*–*V* characteristics of the photodiode under dark, 625 nm (VIS) and 850 nm (NIR) illumination (100 μW cm^−2^) for devices incorporating **a** high-conductivity PEDOT:PSS and **b** low-conductivity PEDOT:PSS interlayer. **c** Steady-state photoresponses under VIS and NIR illumination (100 μW cm^−2^) at discrete biases of −0.5 V, 0 V, and +0.5 V. **d** Steady-state photocurrent as a function of MAPbI_3_ thickness (approx. 50, 100, 250, and 350 nm) under VIS and NIR illumination at ±0.5 V. **e** Photocurrent–irradiance plot under VIS illumination at +0.5 V, showing bipolar behavior with increasing irradiance. **f** Responsivity (*R*_*λ*_) under VIS and NIR illumination at −0.5, 0, and +0.5 V as a function of irradiance (10 nW cm^−2^ to 1 mW cm^−2^)

The current–voltage (*I–V*) characteristics of photodiodes incorporating HCP and LCP interlayers are shown in Fig. 3a, b, respectively. Measurements were performed under dark condition and under illumination at 625 nm (visible) and 850 nm (NIR) at a constant irradiance of 100 μW cm^−2^. Unless otherwise stated, positive bias refers to a positive potential applied to the Au/Ag top electrode while the silicon bottom contact is grounded. Under illumination, the HCP-based device exhibits a clear bias-dependent photoresponse for both wavelengths. At −0.5 V, illumination at both VIS and NIR wavelengths generates photocurrents that exceed the dark current. At +0.5 V, the response becomes strongly wavelength-dependent; the NIR-induced current remains above the dark level, whereas the VIS-induced current is suppressed below it. At 0 V, both wavelengths produce similar photocurrent levels, showing no significant spectral selectivity. Overall, the HCP-based device exhibits large photocurrent magnitudes and a pronounced bias-programmable response within a low operating voltage window (− 0.5, 0, and +0.5 V). In contrast, devices incorporating an LCP interlayer exhibit substantially reduced photocurrents under low-bias conditions, as shown in Fig. 3b (0.1 μA at −0.5 V, two orders of magnitude lower than that of the HCP device).

To further clarify the bias-dependent behavior of the HCP device, steady-state current measurements and spectral response analyses were conducted (Fig. 3c; Figure S4, Supporting Information). The HCP-based device exhibited a substantial photocurrent and a pronounced bias-programmable response within a low operating voltage window of −0.5, 0, and +0.5 V. At −0.5 V, the device showed a relatively stronger photoresponse under NIR illumination than under visible light, whereas comparable photoresponses to both wavelengths were observed at 0 V. Notably, at +0.5 V, the device exhibited bipolar photoresponse behavior, generating positive photocurrent under NIR illumination and negative photocurrent under visible illumination. This bias-tunable spectral response was more clearly confirmed by the spectral response curves shown in Figure S4 (Supporting Information).

In the conventional BPD employing the LCP interlayer, only a single junction remains electrically active under a specific bias polarity, whereas the opposite junction acts as a resistive bottleneck. Under negative bias, the silicon/PEDOT:PSS junction becomes the primary photoactive site, while the MAPbI_3_ layer acts as a passive resistive spacer in series, effectively modulating the overall charge transport through the monolithic stack. Due to the limited conductivity of both the MAPbI_3_ layer and the MAPbI_3_/PEDOT:PSS interface, coupled with the low hole-transporting ability of the LCP interlayer, a significant voltage drop occurs across the MAPbI_3_/PEDOT:PSS region, leaving the bottom silicon junction insufficiently biased for efficient charge extraction.

In contrast, the HCP-based device exhibits fundamentally different behavior. The HCP functions as a low-resistance interlayer, facilitating seamless bias distribution across the stacked junctions. Furthermore, the interactions at the PEDOT:PSS/perovskite interface induce defect-rich regions that act as nonselective leakage pathways, likely consisting of partially degraded perovskite and trap-rich domains (Figure S5, Supporting Information). The formation of defect-rich domains is driven by acidic interactions between PSS and the perovskite, and the interfacial incompatibility of the perovskite solution with the PEDOT:PSS surface [41–46, 63]. Although both LCP- and HCP-based devices exhibit similar interfacial defect formation, the higher doping concentration and superior conductivity of HCP, together with a reduced interfacial energy barrier at the n-Si/PEDOT:PSS junction arising from its lower work function (4.92 eV for HCP vs. 5.09 eV for LCP; Figure S6, Supporting Information), transform these defect regions into effective current pathways.

While such parasitic pathways are generally considered detrimental in conventional perovskite optoelectronics [41–46], they are reconfigured here as functional pathways. This approach enables a superior photoresponse at low operating voltages (< 1 V) by effectively overcoming the resistive-barrier limitations inherent in traditional BPD architectures. To the best of our knowledge, this is the first report of exploiting PEDOT:PSS/perovskite interfacial leakage as an operating principle for high-sensitivity spectral discrimination.

To determine the optimal device configuration, we investigated BPDs with four different MAPbI_3_ thicknesses ranging from 50 to 350 nm (Fig. 3d). The observed monotonic reduction in photocurrent at −0.5 V with increasing perovskite thickness stems from the portion of the MAPbI_3_ layer located above the defect-rich interfacial domains. This unreacted upper region functions as a substantial resistive barrier. At +0.5 V, the devices exhibited bipolar photoresponse, characterized by a weak negative photocurrent under 625 nm illumination and a strong positive photocurrent under 850 nm illumination. Interestingly, as the MAPbI_3_ thickness reached 350 nm, the characteristic negative photocurrent vanished; instead, the device exhibited a stronger photoresponse under 625 nm relative to 850 nm, effectively reverting to the behavior of a standard LCP-based architecture. This transition is attributed not only to the increased resistive barrier of the thicker MAPbI_3_ layer but also to the thickness-dependent optical absorption, consistent with the absorption simulations in Fig. 2c, d. These results demonstrate that an excessively thick MAPbI_3_ layer nullifies the synergistic advantages of the HCP interlayer. Therefore, the MAPbI_3_ thickness must be carefully optimized to balance the bipolar response at 0.5 V with efficient photocurrent extraction at −0.5 V. Accordingly, a 100 nm thick MAPbI_3_ layer was identified as the optimal configuration to satisfy both requirements.

The bipolar photoresponse of the BPD with a 100 nm perovskite layer was evaluated at +0.5 V as a function of irradiance (Fig. 3e). Notably, under 625 nm illumination, the photocurrent exhibited a distinctive nonlinear evolution, wherein the initially negative photocurrent underwent a polarity reversal once the incident irradiance surpassed the threshold of 100 μW cm^−2^. This behavior stands in sharp contrast to the response under 850 nm (Figure S7, Supporting Information), which exhibits a monotonic increase in photocurrent with increasing irradiance.

The wavelength- and bias-dependent nature of the photoresponse is more clearly revealed in the responsivity curves. Responsivity (*R*_*λ*_) was evaluated as a function of irradiance (10 nW cm^−2^ to 1 mW cm^−2^) under VIS (625 nm) and NIR (850 nm) illumination at −0.5, 0, and +0.5 V (Fig. 3f; Figure S8, Equation S1, Supporting Information). At −0.5 V, the device showed high and nearly constant responsivity over the entire irradiance range under both VIS and NIR illumination, with peak values of *R*_625_ ≈ 0.67 A W^−1^ and *R*_850_ ≈ 1.1 A W^−1^. Such behavior signifies efficient collection of photogenerated carriers across a broad dynamic range, facilitated by the HCP interlayer.

As the applied bias approaches 0 V and +0.5 V, the responsivity becomes increasingly sensitive to irradiance. At 0 V, both VIS and NIR responsivities decrease at higher irradiance, which is consistent with the reduced internal electric field and the augmented probability of carrier recombination under zero-bias operation. At +0.5 V, the nonlinear behavior becomes profoundly wavelength-dependent. The NIR responsivity remains relatively stable with irradiance, whereas the VIS responsivity decreases by approximately 90% within the nonlinear regime and then rises again once the irradiance exceeds the polarity-transition threshold of 100 μW cm^−2^, as shown in Fig. 3e. This distinctive behavior represents an unusual nonlinear photoresponse rarely observed in conventional BPDs or other photodiodes. Since this nonlinear characteristic serves as the fundamental mechanism for the wavelength discrimination, the device is hereafter termed a nonlinear BPD (NL-BPD).

To further quantify the bias-dependent linearity, the photocurrent magnitude was analyzed as a function of irradiance over the same measurement range (Figure S8, Equation S2, Supporting Information). Consistent with the responsivity trends, both VIS and NIR responses increased nearly proportionally with irradiance at −0.5 and 0 V, with slopes of 0.97 and 0.96 at −0.5 V, and 0.96 and 0.94 at 0 V for the VIS and NIR responses, respectively, yielding LDRs of ≥ 100 dB at −0.5 V and ≈ 60 dB at 0 V for both wavelengths [64]. Since the −0.5 V responses remained linear throughout the irradiance range examined, the LDR is conservatively evaluated as at least 100 dB, providing sufficient linearity for the targeted measurements. At +0.5 V, the NIR response remained monotonic with irradiance, exhibiting a slope of 1.11 over a measured dynamic range of ≈ 74 dB. In contrast, the nonlinear polarity transition of the VIS response (Fig. 3e) precluded the assignment of a well-defined LDR value. This behavior indicates that the VIS photoresponse can be electrically reconfigured, shifting from linear intensity quantification under negative bias to nonlinear spectral discrimination under positive bias.

Specific detectivity (D^*^_λ_) values were calculated using a shot-noise-limited model (Figure S9, Equation S3, Supporting Information) [65]. Under −0.5 V bias, the device achieves peak detectivities of D^*^_625_ ≈ 6.4×10^11^ Jones and D^*^_850_ ≈ 1.1×10^12^ Jones. At +0.5 V, detectivity remains on the order of ~10^10^ Jones for both wavelengths with the discernible reduction and subsequent recovery under VIS illumination within the nonlinear region. This behavior is consistent with the responsivity trends discussed above. The transient photoresponse was further characterized by extracting rise and decay times under pulsed illumination (Figure S10, Supporting Information). At −0.5 V, the VIS and NIR responses exhibited rise/decay times of approximately 5.1/12 μs and 2.8/99 μs, respectively. Under +0.5 V bias, the NIR response exhibited rise and decay times of 120 μs and 99 μs, respectively. These response speeds are sufficient for recording physiological signals, as demonstrated in the following section. The operational repeatability was measured under light on/off cycling (Figure S11, Supporting Information). The photocurrent remains stable over 100 cycles (500 s) with no observable degradation or drift, indicating a robust and reproducible photoresponse. To assess long-term operational stability, the photoresponse of the device under VIS and NIR illumination was monitored for 10 days, as shown in Figure S12 (Supporting Information).

Overall, the NL-BPD configuration enables stable, fast, and tunable photoresponses even under sub-volt (0.5 V) bias conditions. The enhanced charge transport environment, facilitated by highly conductive interfacial pathways, allows for systematic tuning of response linearity via the applied bias. This architecture promotes transitions among linear, quasi-nonlinear, and super-nonlinear regimes, providing expanded operational degrees of freedom that are unattainable with conventional monolithic structures. As summarized in Table S1, compared with previously reported bias-modulated dual-band photodetectors, the NL-BPD notably combines low-voltage operation with bias-programmable linear to nonlinear response modes while maintaining competitive responsivity, detectivity, and dynamic range. The observed photoresponse behaviors confirm that such device-level reconfiguration is a pivotal strategy for achieving programmable photodetection.

### Operating mechanism of NL-BPD and control devices

**Fig. 4:**
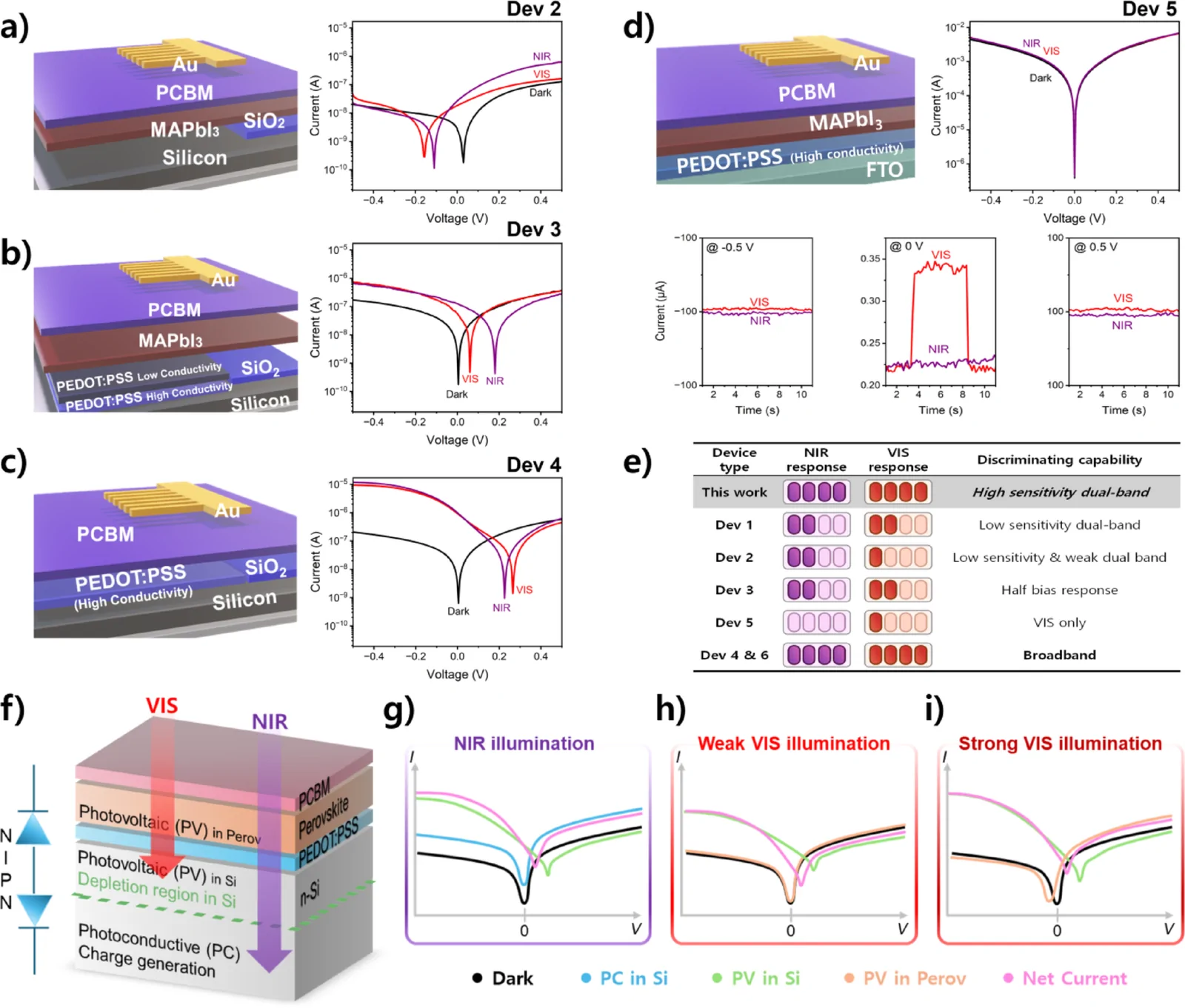
Control-device analysis and operating mechanism of the NL-BPD. **a**–**d** Schematic device structures and corresponding *I*–*V* characteristics of **a** Au/PCBM/MAPbI_3_/Si, **b** Au/PCBM/MAPbI_3_/LCP/HCP/Si, **c** Au/PCBM/HCP/Si, and **d** Au/PCBM/MAPbI_3_/HCP/FTO on glass. **e** Comparative summary of spectral discrimination capability and wavelength-dependent response for the NL-BPD (this work) and control devices (Dev 1–6). Detailed characteristics are provided in Fig. 3a (NL-BPD), 3b (Dev 1), 4a (Dev 2), 4b (Dev 3), 4c (Dev 4), 4d (Dev 5), and Figure S13 (Dev 6). **f** Schematic of the NP^+^IN monolithic stack, illustrating photovoltaic (PV) and photoconductive (PC) contributions under red and NIR excitation. **g**–**i** Schematic *I–V* curves illustrating the proposed mechanism under (**g**) NIR illumination, **h** weak VIS illumination, and **i** strong VIS illumination. The legend is common to all panels (**g**–**i**)

A series of control devices (Dev 2–6) with modified structural configurations was fabricated and comparatively analyzed with reference to the optimized NL-BPD (this work), as detailed in Fig. 4a–d and Figure S13 (Supporting Information). This approach was used to clarify the operating mechanism of the proposed NL-BPD architecture and the functional roles of the individual layers. First, the role of the HCP interlayer was investigated using a device without the interlayer (Au/PCBM/MAPbI_3_/Si, Dev 2). As shown in Fig. 4a, removal of the interlayer inhibits the formation of the leakage-mediated carrier-transport pathways associated with the interfacial reaction between PEDOT:PSS and MAPbI_3_, leading to markedly suppressed photocurrent relative to the NL-BPD. In particular, the negligible photocurrent at −0.5 V in Dev 2 indicates that carriers generated in n-Si were predominantly unable to traverse the upper resistive MAPbI_3_ layer. The importance of a highly conductive interface was further examined using Dev 3, featuring a stacked LCP/HCP layer where the MAPbI_3_ layer is in direct contact with the LCP layer (Fig. 4b). The photoresponses of Dev 3 at both −0.5 and +0.5 V, exhibiting low and similar photocurrent under VIS and NIR, closely mirror those of the LCP-only device (Dev 1), underscoring that high photoresponsivity and bias-dependent spectral discrimination are both contingent upon the direct coupling of the defect-rich perovskite interface with a low-resistance contact (HCP).

To elucidate the fundamental role of the dual-photoactive-layer architecture—comprising an MAPbI_3_ VIS absorber and an n-Si NIR absorber—in achieving spectral discrimination, we conducted a comparative study against single-absorber control devices. The perovskite-free structures (Dev 4: Au/PCBM/HCP/Si and Dev 6: Au/HCP/Si) exhibited a typical broadband photoresponse without spectral selectivity [38, 66, 67], as shown in Fig. 4c and Figure S13 (Supporting Information). In contrast, replacing the silicon substrate with a transparent FTO/glass electrode (Dev 5) effectively eliminated the NIR response while preserving visible-light sensitivity (Fig. 4d).

These results confirm that the integration of dual photoactive layers is indispensable for achieving spectral discrimination. A pivotal characteristic that distinguishes the NL-BPD from conventional perovskite PIN diodes or solar cells is its pronounced leakage current. While typically viewed as a detrimental factor [63], this high dark current is strategically harnessed as a fundamental operating mechanism in this work to enable nonlinear photodetection. Consequently, the NL-BPD architecture stands alone in its ability to simultaneously achieve high-sensitivity dual-band detection and bias-tunable response modulation at a low operating voltage window (± 0.5 V, Fig. 4e).

In semiconducting photodetectors, photocurrent is generally generated through two primary mechanisms: the photovoltaic (PV) effect and the photoconductive (PC) effect. The PV effect originates from built-in-field-driven carrier separation at a photoactive junction, whereas the PC effect arises from illumination-induced conductivity enhancement in the bulk under an applied bias (Figure S14, Note S1, Supporting Information). In the NL-BPD, these two mechanisms are activated differently depending on the optical penetration depth and the region in which the incident light is absorbed (Fig. 4f). As NIR light penetrates deeply into the n-Si layer, it generates both a PV current at the Si/HCP junction and a PC current within the Si bulk. In contrast, visible light—owing to its shallower penetration depth—is absorbed primarily in the perovskite layer. Consequently, it mainly modulates the competing PV currents generated at the Si/HCP and perovskite/HCP junctions [66, 68, 69].

Under NIR illumination, the photoresponse is governed by the synergistic action of the PV and PC mechanisms, as illustrated in Fig. 4g. At negative bias, the Si/HCP junction is reverse-biased, so the built-in electric field efficiently separates photogenerated electron–hole pairs and produces a robust PV photocurrent. At positive bias, however, the applied field counteracts the built-in potential at the Si/HCP junction, thereby attenuating the PV contribution and causing it to oppose the dark current. If this PV component were the only contribution, the total current would decrease slightly (relative to dark current) under illumination, as represented by the green line in Fig. 4g. Experimentally, however, the measured net current remains higher than this ideal PV-only response (green line) as NIR absorption in bulk Si simultaneously yields a PC contribution (blue line). Under positive bias, the PC current flows in the same direction as the dark current, thereby compensating for the reduced PV component and resulting in a net increase in the total current.

The visible-light response is more complex because the thin perovskite layer allows part of the incident light to reach the underlying Si, and VIS photons have sufficient energy to excite both MAPbI_3_ and Si. Because the two junctions form a back-to-back configuration, their built-in electric fields are oppositely oriented, giving rise to two competing PV photocurrent components with opposite polarities (Fig. 4h, i; Figure S14, Supporting Information). Under negative bias, the reverse-biased Si/HCP junction dominates, resulting in a VIS response similar to that observed under NIR illumination. Under positive bias, in contrast, the reverse-biased perovskite/HCP junction drives photocurrent in the direction of the applied bias. Although the Si/HCP junction is forward-biased at +0.5 V, the applied bias remains well below its built-in potential (Figure S14), so the residual built-in field still separates photogenerated carriers in Si and yields a substantial PV current that opposes the perovskite/HCP contribution.

At this bias, the relative strengths of the two PV contributions vary with irradiance, giving rise to the characteristic nonlinear VIS response. As discussed above, the interaction between MAPbI_3_ and highly conductive PEDOT:PSS (HCP) creates a defect-rich interfacial region that promotes rapid carrier recombination and generally reduces charge-collection efficiency at the perovskite/HCP junction (Fig. 4d) [63]. Under weak visible illumination (Fig. 4h), this recombination severely suppresses the perovskite-side PV contribution, allowing the Si/HCP junction to remain dominant; consequently, the net photocurrent remains below the dark current level. Under strong visible illumination (Fig. 4i), however, carrier generation in the perovskite becomes sufficiently large enough to overcome the recombination losses, and the PV current from the perovskite/HCP junction eventually exceeds that from the Si/HCP junction. This crossover triggers a reversal in the sign of the net photocurrent, producing the polarity switching observed in Fig. 3e.

Overall, the nonlinear and spectral-dependent response of the NL-BPD originates from the highly doped PEDOT:PSS layer, which induces asymmetric recombination dynamics at the two junctions. This asymmetry selectively suppresses the perovskite-side contribution under low-intensity visible illumination but allows it to emerge at higher irradiance; this transition gives rise to the characteristic nonlinear responsivity and enhanced photodetection performance illustrated in Fig. 3a.

### Bias-programmable spectral decoding: pulse oximetry and NDVI demonstrations

The NL-BPD facilitates the electrical decoupling of mixed VIS–NIR signals under low-light intensities through its bias-programmable functionality. This robust selectivity is particularly advantageous for detecting weak, spectrally overlapping signals inherent in biomedical diagnostics and precision agriculture [70–72]. To demonstrate its practical utility, we evaluated the system-level performance in two optical sensing geometries: transmission-mode single-point pulse oximetry and reflection-mode NDVI-based crop monitoring.

**Fig. 5:**
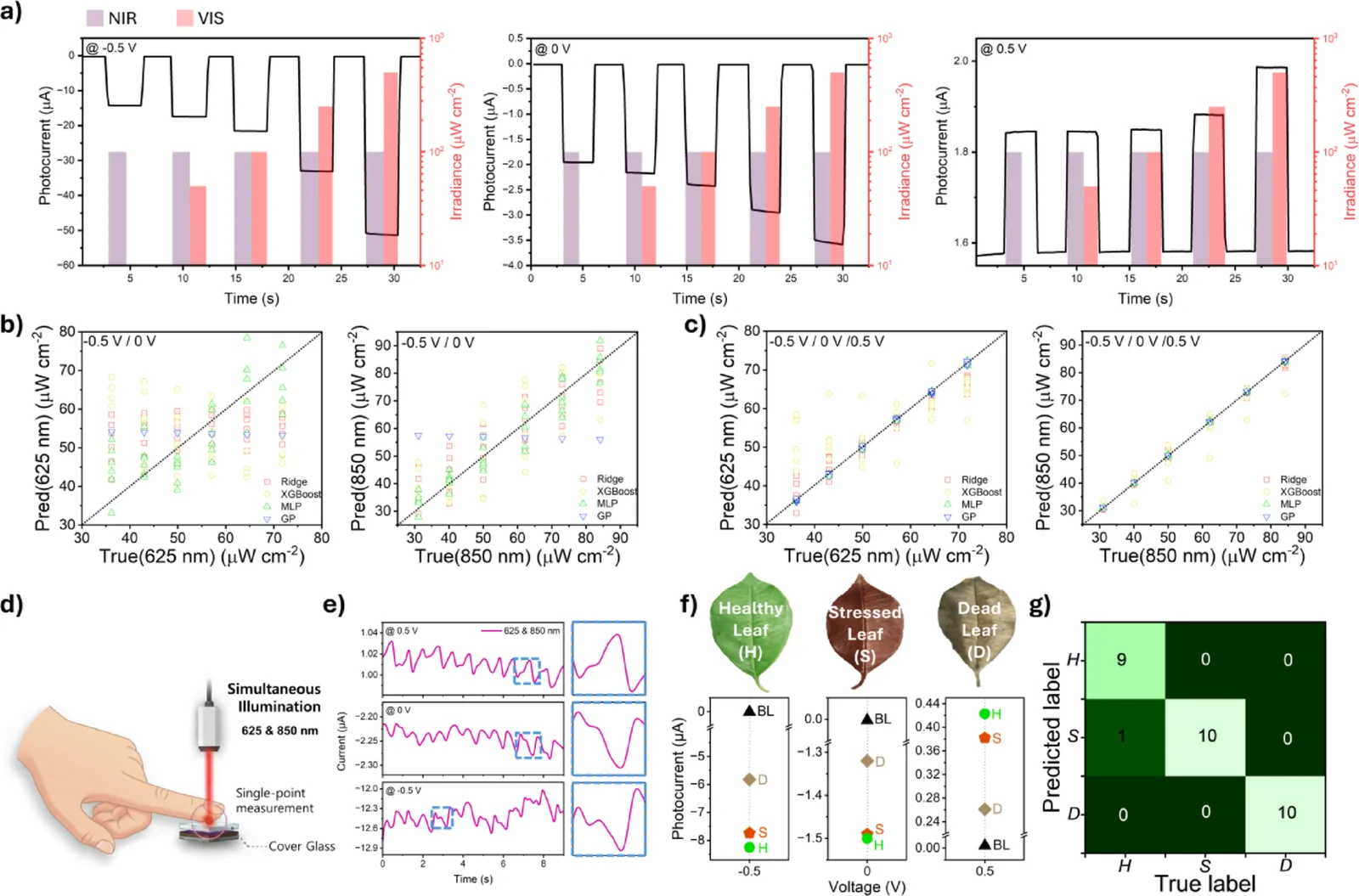
Bias-programmable spectral decoding and multispectral sensing applications. **a** Time-resolved photocurrent traces recorded at different operating biases (− 0.5 V, 0 V, and 0.5 V) under simultaneous VIS and NIR illumination pulses with varying irradiances. The NIR irradiance was fixed at 100 μW cm^−2^ while the VIS irradiance was modulated from 10 to 500 μW cm^−2^. **b** Parity plots showing predicted versus true irradiance for the 625 nm and 850 nm channels, using two-bias photocurrent inputs (− 0.5 V, 0 V). **c** Corresponding parity plots obtained using three-bias inputs (− 0.5 V, 0 V, 0.5 V), showing markedly improved agreement with the identity line (dotted). **d** Experimental setup for single-point pulse oximetry. **e** Real-time PPG waveforms obtained at different operating biases (− 0.5 V, 0 V, 0.5 V) under ambient conditions. **f** Representative photographs of leaf samples categorized as healthy (H), stressed (S), and dead (D), with the corresponding steady-state photocurrents measured at −0.5 V, 0 V, and 0.5 V under simultaneous visible and NIR illumination. A baseline (BL) measured without samples is included for reference. **g** Confusion matrix illustrating the classification accuracy of the Gaussian process (GP) model

Practical signal reconstruction performance was evaluated using data-driven algorithms tailored to each application. To enable pulse oximetry with a filter-free architecture, the encoding capability of the device under mixed illumination was assessed by measuring steady-state photocurrents as a function of incident irradiance (10–500 μW cm^−2^) under simultaneous VIS and NIR illumination (Fig. 5a). When the NIR irradiance was fixed at 100 μW cm^−2^ while the VIS irradiance was varied, additive photoresponses were observed at biases of −0.5 V and 0 V. Under these conditions, the total photocurrent increased monotonically with the intensities of both light sources. In contrast, qualitatively distinct behavior emerged at +0.5 V bias, introducing an additional degree of freedom for wavelength discrimination. In the low-illumination regime below ≈100 μW cm^−2^, the VIS-induced current contributes negligibly owing to its suppressed response, as observed in Fig. 3e. Therefore, the total photocurrent remains nearly identical to that extracted under NIR-only illumination regardless of VIS irradiance. To further support this behavior, we also investigated the photocurrent traces under a fixed VIS irradiance (100 μW cm^−2^) while varying the NIR irradiance from 10 to 500 μW cm^−2^ (Figure S15, Supporting Information). The photoresponses at −0.5 V and 0 V exhibited monotonic and additive characteristics, whereas the response at +0.5 V predominantly followed the NIR-induced signals across the entire intensity range.

A database was established to extend the multi-bias spectral encoding strategy toward practical signal reconstruction (Table S2, Supporting Information). On the basis of this dataset, four machine-learning algorithms—Ridge regression, XGBoost, multilayer perceptron (MLP), and Gaussian process (GP)—were employed to construct regression models (Figure S16, Supporting Information). Model performance was evaluated using the coefficient of determination (R^2^) and the mean absolute error (MAE). When only the −0.5 V and 0 V bias responses were used as input variables (Fig. 5b), all models showed broad dispersion and failed to converge toward the identity line. Under these two-bias conditions, the MLP model showed the best performance, yielding an average R^2^ of 0.7483 and an average MAE of 5.53 μW cm^−2^. In striking contrast, when the +0.5 V response was incorporated to include all three bias conditions as inputs (Fig. 5c), predictions from all four models clustered tightly around the identity line. In particular, the relatively simple MLP and GP models exhibited near-ideal agreement between the predicted and actual values. Utilizing the GP model with three-dimensional bias inputs (*I*_−0.5V_, *I*_0V_, and *I*_+0.5V_), R^2^ values of 0.9993 and 0.9999 were achieved for the 625 and 850 nm channels, respectively, corresponding to an exceptional average R^2^ of 0.9996 and a remarkably low average MAE of 0.20 μW cm^−2^. This performance represents an approximately 28-fold improvement in accuracy over the two-dimensional input case (*I*_−0.5V_ and *I*_0V_). Moreover, when the +0.5 V signal was randomly permuted (Figure S17, Supporting Information), the predictive performance degraded markedly, with an average R^2^ of 0.3719 and an average MAE of 8.97 μW cm^−2^. This result confirms that the performance enhancement stems from the physically intrinsic information encoded at +0.5 V, rather than the mere addition of an extra numerical dimension.

Building on this algorithmic framework, the spectral reconstruction capability of the NL-BPD was further validated through practical physiological measurements. Photoplethysmography (PPG) signals were acquired under simultaneous 625 nm and 850 nm illumination (Fig. 5d, e), where stable PPG waveforms were successfully recorded under all three bias conditions. A conventional non-inverted PPG waveform was observed at +0.5 V, whereas inverted waveforms were obtained at −0.5 V and 0 V, reflecting the reversal of photocurrent polarity relative to that at +0.5 V. The reliability of these PPG signals under mixed illumination was further verified through single-wavelength control experiments and long-term continuous measurements (Figure S18, Supporting Information), demonstrating the stability and reproducibility of the platform.

Using the PPG waveforms, pulse rate was determined from the temporal periodicity (Equation S4, Supporting Information). The measured values of 86, 80, and 73 bpm at −0.5 V, 0 V, and +0.5 V bias, respectively, fell within the reference range recorded by the commercial oximeter (70–92 bpm) (Movie S1–S3, Supporting Information). Peripheral oxygen saturation (SpO_2_) was calculated using the modified Beer-Lambert law (Equation S6, Supporting Information) in conjunction with tabulated molar extinction coefficients for HbO_2_ and Hb obtained from the Prahl hemoglobin absorption database (Table S3, Note S2, Supporting Information) [73, 74]. A modulation ratio *R* of 0.899, derived from reconstructed VIS and NIR optical intensities, yielded an SpO_2_ value of 95.4%. This value shows reasonable agreement with the 98–100% range measured simultaneously using a commercial fingertip pulse oximeter (Movie S1–S3, Supporting Information). Additional calibration and clinical validation are required to address the limitations of the modified Beer–Lambert approach. The AC/DC-based modulation ratio may be affected by tissue scattering, wavelength-dependent variations in effective optical path length, and uncertainties in reconstructed VIS–NIR signals, as errors in extracting the pulsatile AC amplitudes and DC baselines can alter the calculated ratio. These limitations also carry over to subsequent SpO_2_ estimation, which is further subject to uncertainties associated with the use of tabulated hemoglobin extinction coefficients without empirical calibration [51]. Nevertheless, it is noteworthy that, as a proof of concept, this work presents a monolithic, filter-free architecture capable of real-time SpO_2_ monitoring by decoupling superimposed spectral signals within a single integrated detector.

NDVI measurements were also performed using the same monolithic photodetector (Figure S19, Note S3, Supporting Information). Figure 5f compares steady-state photocurrents measured under mixed illumination for three representative samples: healthy (H), stressed (S), and dead (D) leaves. Background light unrelated to the sample signal was measured independently and utilized as the experimental baseline (BL). The healthy leaf exhibited the highest photocurrent across all bias conditions, primarily due to the inherent optical characteristics of healthy vegetation: its high NIR reflectance coupled with strong absorption of red light. In contrast, stressed and dead leaves showed progressively reduced photocurrents. These reductions arise from degraded reflectance associated with changes in tissue structure and pigment composition [75]. Photocurrent responses obtained under single-wavelength illumination at 625 and 850 nm (Figure S20, Supporting Information) further validate the reliability and precision of the simultaneous dual-wavelength sensing system. While the absolute photocurrents for each leaf at individual wavelengths may exhibit complex variations, the normalized difference between NIR and visible photocurrents follows NDVI trends.

Precise isolation of purely reflected light from leaf surfaces was not possible under laboratory conditions. Contributions from scattered light and ambient optical variations could not be completely eliminated; therefore, a GP-based health status prediction model was implemented (Table S4, Table S5, Supporting Information). A Gaussian process classifier was used to construct a model capable of predicting leaf health status using photocurrent responses measured under three bias conditions. The model was trained using the multi-bias photoresponses as inputs and NDVI-based health states as outputs. Measurements were obtained at different positions across 30 leaf samples, comprising 10 healthy, 10 stressed, and 10 dead samples. Classification performance was evaluated using leave-one-out cross-validation (LOOCV), in which each sample was used once for testing while the remaining samples were used for training. As shown in the confusion matrix (Fig. 5g), the model successfully predicted the health status for 29 out of 30 samples, achieving an exceptional classification accuracy exceeding 96%. While the limited dataset from a single plant species may restrict the generalizability of these results, requiring larger and more diverse datasets and refined health classification schemes for practical commercialization, this study successfully demonstrates the proof-of-concept feasibility of plant health monitoring using a single sensor platform.

Collectively, these results confirm that accurate classification across physiological and vegetation sensing tasks arises primarily from device-level spectral encoding rather than algorithmic complexity. The proposed filter-free configuration, therefore, represents a compact and integrated sensing platform for spectrally resolved biosensing and agro-environmental applications.

## Conclusions

In this work, we present a monolithic NL-BPD that enables filter-free, single-pixel discrimination between VIS and NIR light under both low-voltage and low-light conditions. The integration of a ~100 nm MAPbI_3_ perovskite absorber with an HCP interlayer intentionally induces a partial interfacial reaction, creating defect-mediated charge transport pathways at the interface and within the upper perovskite. Coupled with the optimized perovskite thickness, these pathways facilitate the simultaneous photoactivation of both perovskite and Si absorbers, ensuring seamless carrier transport from the bottom Si junction to the top electrode. Consequently, cooperative charge extraction from both absorbers is achieved under sub-volt operation (± 0.5 V). This structural reconfiguration enables two distinct, bias-defined operating modes: a wide-linear-range detection mode (LDR ≥ 100 dB) at −0.5 V and a spectral-discrimination mode at +0.5 V, where the NIR response maintains a consistent polarity with relatively smooth irradiance dependence, whereas the visible response evolves from linear to strongly nonlinear. Notably, at an irradiance of 100 μW cm^−2^, the visible response undergoes a distinct polarity inversion at +0.5 V. This voltage-controlled transition from a linear to a bipolar photoresponse generates an intrinsic electrical spectral fingerprint, enabling computational demultiplexing of mixed VIS–NIR signals using a single detector without optical filters or spatially separated pixels.

Combining this device-encoded spectral diversity with machine-learning-based decoding enables accurate reconstruction of mixed VIS–NIR irradiances (average R^2^ = 0.9996) and single-point pulse oximetry under mixed illumination, as well as NDVI-based vegetation analysis with an accuracy exceeding 96% using a Gaussian process classification model. Overall, this work establishes a device-level strategy for programmable multispectral photodetection by engineering charge-transport and bias-distribution in a monolithic stacked junction. This compact, low-power platform introduces a new paradigm for filter-free single-pixel spectral sensing and provides a foundation for integrated photonic systems in wearable healthcare, smart agriculture, and autonomous environmental monitoring.

## Experimental section

### Materials

PEDOT:PSS solutions (PH1000 and AI4083) were purchased from Ossila. Methylammonium iodide (MAI, 99%), phenyl-C_61_-butyric acid methyl ester (PCBM, 99%), dimethyl sulfoxide (DMSO, anhydrous), N,N-dimethylformamide (DMF, anhydrous), and chlorobenzene (CBZ, anhydrous) were used as received from Sigma-Aldrich. Lead(II) iodide (PbI_2_, 99.99%) was purchased from Thermo Fisher Scientific. Silicon wafers with a 100 nm SiO_2_ layer (< 100>, n-type, 1–10 Ω·cm) were purchased from iTASCO. Buffered oxide etchant (BOE, 6:1) was obtained from Samchun Chemical. Acetone and isopropyl alcohol (IPA) were purchased from DUKSAN. Epoxy resin (AR-16) was purchased from EPONS.

### Device fabrication

N-type silicon substrates (1.3 × 1.3 cm^2^) were prepared through sequential cleaning in acetone and IPA for 5 min each. To expose the bottom contact area, two-thirds of the SiO_2_ layer was selectively removed using BOE as the etchant and Kapton tape as a protective mask. After etching, the substrates were rinsed and subjected to a second cleaning cycle in deionized (DI) water, acetone, and IPA. UV-ozone treatment was then applied for 15 min to improve surface wettability. PEDOT:PSS was spin-coated at 5000 rpm for 40 s and annealed at 150 °C for 15 min. The MAPbI_3_ absorber layer was formed by spin-coating 75 μL of a 0.5 m precursor solution (MAI:PbI_2_ = 1:1 in DMSO:DMF, 1:4 v/v) at 5000 rpm for 30 s. During the spinning process, 300 μL of chlorobenzene (CBZ) anti-solvent was rapidly dispensed onto the spinning substrate 8 s after the start of the spin cycle. The resulting film was annealed at 100 °C for 20 min. Then, the PCBM electron-transport layer (20 mg mL^−1^ in CBZ) was spin-coated at 2000 rpm for 40 s and annealed at 100 °C for 10 min. A top electrode stack consisting of Au (10 nm) and Ag (55 nm) was deposited via thermal evaporation at 0.5 Å s^−1^ through a grid-type shadow mask. The effective active area of the device, defined by the Au/Ag grid electrode, was 8 mm^2^. Finally, the active area was encapsulated with a 0.1 mm-thick glass slide secured with epoxy resin (cured for 24 h). Cu foil was electrically connected to the backside of the Si substrate through silver paste. All steps after PEDOT:PSS deposition and before encapsulation were performed in a nitrogen-filled glovebox.

### Film characterization

The thickness of the perovskite films was measured using a stylus profilometer (DektakXT, Bruker). Crystallographic analysis was performed by X-ray diffraction (XRD, Rigaku Ru-200B) using a CuKα source (40 kV and 100 mA). Optical absorption spectra were measured using a UV–vis spectrometer (AvaSpec-ULS2048L-USB2, Avantes). The work function of the PEDOT:PSS layer was measured by ultraviolet photoelectron spectroscopy (UPS, NEXSA). For microstructural and compositional analysis, cross-sectional specimens were prepared using a focused ion beam (FIB) system (NX5000, Hitachi). High-angle annular dark-field scanning transmission electron microscopy (HAADF-STEM) images were subsequently acquired using a Tecnai G2 F30 S-Twin microscope operated at an accelerating voltage of 300 kV. Elemental mapping was performed using energy-dispersive X-ray spectroscopy (EDS) with an energy resolution of 136 eV.

### Device characterization

*I–V* characteristics of the photodetector were measured using a Keithley 4200 source meter under illumination from discrete wavelength sources (625 and 850 nm, Mightex). Spectral photoresponse from 400 to 1000 nm was measured using a monochromator system (Zolix). Rise and decay times were measured using a digital oscilloscope (Tektronix, DPO4014B) connected to a low-noise current preamplifier (Stanford Research Systems, SR570). Photocurrent stability and repeatability were evaluated using a Keithley 2612B source meter under pulsed LED illumination (Mightex). All electrical measurements were conducted under ambient conditions in a dark box to minimize noise and stray light.

### Application testing

To demonstrate the device's utility for biometric monitoring, single-point photoplethysmography (PPG) and oximetry measurements were performed under controlled conditions. The index finger of a healthy adult volunteer was positioned over the device's active area, separated by a glass passivation layer to maintain interfacial integrity. Under simultaneous illumination at 625 and 850 nm, PPG-containing photocurrent signals were recorded in a transmission configuration using a Keithley 4200 source meter.

For vegetation health analysis, leaf samples were collected from the Gwangju Institute of Science and Technology (GIST) campus garden. To minimize biological variation, all leaves were obtained from the same plant species. The samples were categorized into three physiological states based on their chlorophyll-related optical signatures: "healthy" (turgid, green foliage with high chlorophyll content), "stressed" (early-stage discoloration with reduced hydration), and "dead" (dried, brown tissue with depleted chlorophyll and structural degradation, collected from the ground). Photoresponses were recorded using a Keithley 4200 source meter in a reflectance configuration under simultaneous visible and NIR illumination to emulate real-world monitoring conditions.

## Supplementary Information

Supplementary Material 1

Supplementary Material 2

Supplementary Material 3

Supplementary Material 4

## Data Availability

The data that support the findings of this study are available from the corresponding author upon reasonable request.

## Abbreviations

BL: Baseline
BOE: Buffered oxide etchant
BPD: Back-to-back photodiode
CBZ: Chlorobenzene
DI: Deionized
DMF: *N*,*N*-dimethylformamide
DMSO: Dimethyl sulfoxide
EDS: Energy-dispersive X-ray spectroscopy
FIB: Focused ion beam
FTO: Fluorine-doped tin oxide
GP: Gaussian process
HAADF: High-angle annular dark-field
HCP: High-conductivity PEDOT:PSS
IPA: Isopropyl alcohol
I–V: Current–voltage
LCP: Low-conductivity PEDOT:PSS
LDR: Linear dynamic range
LED: Light-emitting diode
MAE: Mean absolute error
MAI: Methylammonium iodide
MAPbI_3_: Methylammonium lead iodide
MLP: Multilayer perceptron
NDVI: Normalized difference vegetation index
NIR: Near-infrared
NL-BPD: Nonlinear back-to-back photodiode
OPD: Organic photodetector
PC: Photoconductive
PCBM: Phenyl-C_61_-butyric acid methyl ester
PEDOT:PSS: Poly(3,4-ethylenedioxythiophene):poly(styrenesulfonate)
PPG: Photoplethysmography
PV: Photovoltaic
SpO_2_: Peripheral oxygen saturation
STEM: Scanning transmission electron microscopy
UPS: Ultraviolet photoelectron spectroscopy
VIS: Visible
XGBoost: Extreme gradient boosting
XRD: X-ray diffraction
