# Supplementary material for: Bias-programmable linear-to-nonlinear photoresponse for filter-free single-pixel VIS–NIR discrimination
Source: Nano Converg. 2026 Oct 26;13:52. doi: 10.1186/s40580-026-00585-7 (PMC13710738; doi:10.1186/s40580-026-00585-7)
Supplement: Supplementary file 1 [file 40580_2026_585_MOESM1_ESM.docx]

Supporting Information

**Bias-Programmable Linear-to-Nonlinear Photoresponse for Filter-Free Single-Pixel VIS–NIR Discrimination**

*Jiyoon Shin^+^, Masroor Alam^+^, Jaeyeon Kim, Yong Geon Lee, Min Kyu Jeon, Hyeonghun Kim,^*^ and Gun Young Jung^*^*

J. Shin, M. Alam, Y. G. Lee, Prof. G. Y. Jung

Department of Materials Science and Engineering (DMSE), Gwangju Institute of Science and Technology (GIST), Gwangju 61005, Republic of Korea

E-mail: gyjung@gist.ac.kr

J. Kim

Center for Frontier AI Research (CFAR), Agency for Science, Technology and Research (A*STAR), Singapore, 138632 Singapore

M. K. Jeon, Prof. H. Kim

School of Chemical Engineering (SCE), Chonnam National University (CNU), Gwangju 61186, Republic of Korea

E-mail: hyhkim1210@jnu.ac.kr

^+^ These authors contributed equally to this work.

^*^ Corresponding authors.

**Figure S1. Comparison of conventional and monolithic sensing architectures for pulse oximetry and NDVI measurements.** Conventional noninvasive pulse and blood oxygen saturation monitoring systems typically rely on sequentially pulsed multiple light sources, which inherently introduces spatial mismatch and temporal synchronization errors (Δt).^[S1–S4]^ Similarly, NDVI systems often employ spatially separated wavelength-specific detectors or optical filter assemblies, which may limit spectral comparability at an identical sensing location.^[S5,S6]^ In contrast, the proposed monolithic photodiode enables simultaneous acquisition of both wavelength components at the same spatial position within a single-pixel architecture, thereby minimizing the inherent errors associated with conventional discrete-component systems. This configuration allows computational separation of mixed VIS–NIR signals without the need for time-multiplexed illumination or external optical filters. Consequently, this spatially co-located detection strategy reduces alignment complexity and provides a compact platform for integrated bio-environmental sensing.

**Figure S2. X-ray diffraction (XRD) of the MAPbI_3_ perovskite layer.** The diffraction pattern shows dominant peaks at ~14.1°, ~28.4°, and ~31.7°, corresponding to the (110), (220), and (310) planes of tetragonal MAPbI_3_ perovskite phase (CH_3_NH_3_PbI_3_), consistent with established reference patterns for MAPbI_3_ films.^[S7]^

**Figure S3. Optical characterization of MAPbI_3_ perovskite layer.** a) UV–Vis absorption spectrum of a ~100 nm MAPbI_3_ film. b) Tauc plot derived from the absorption spectrum (Figure S3a), indicating an optical bandgap of 1.59 eV.

**Figure S4. Spectral response of the photodiode** measured from 400 to 1000 nm under applied biases of –0.5 V, 0 V, and 0.5 V.

The spectral response of the photodiode was measured from 400 to 1000 nm to confirm that the bias-tunable selectivity is maintained across a broad spectral range. Although the device exhibits broadband functionality, 625 nm (VIS) and 850 nm (NIR) were selected due to their distinct absorption and reflectance contrast, enabling pulse oximetry and NDVI measurements.^[S8,S9]^ While 850 nm is less commonly adopted as a standard clinical wavelength used in pulse oximetry, it serves as a convenient laboratory-accessible NIR source for proof-of-concept demonstrations and enables reliable physiological signal extraction and vegetation-state discrimination within the single-pixel sensing framework. Consequently, these two wavelengths were utilized as the primary optical probes for the optoelectronic characterization in this study.

**Figure S5. TEM image of PEDOT:PSS/MAPbI_3_ interface.** Interfacial interactions between PSS and MAPbI_3_ induce defect-rich reaction regions. These regions create nonselective current pathways.^[S10–S14]^ The image is digitally colorized for clarity, with orange, blue, and green representing MAPbI_3_, HCP PEDOT:PSS, and the reaction region, respectively.

**Figure S6. UPS measurement of PEDOT:PSS films.** Ultraviolet photoelectron spectroscopy (UPS) spectra comparing the work function of high-conductivity and low-conductivity PEDOT:PSS films (HCP and LCP). The secondary electron cutoff reveals work functions of 4.92 eV and 5.09 eV for the HCP and LCP, respectively.^[S15]^

The shift toward a lower work function in the HCP (4.92 eV) compared to the LCP (5.09 eV) originates from the higher effective doping concentration and quasi-metallic character of the HCP layer. This electronic modulation reduces series resistance and facilitates efficient carrier extraction at the interface. Consequently, the HCP acts as a functional interlayer that enables cooperative charge extraction across the stacked absorbers, supporting high-performance operation under low-bias conditions.^[S15–S17]^

**Figure S7. Photocurrent–irradiance plot under NIR.** Monotonic non-bipolar behavior observed at 0.5 V under NIR (850 nm) illumination.

**Figure S8. Linear dynamic range (LDR) of the NL-BPD.** Absolute photocurrent as a function of incident irradiance under VIS (625 nm) and NIR (850 nm) illumination at (a) −0.5 V, (b) 0 V, and (c) +0.5 V. Solid lines are power-law fits, with the extracted slopes and corresponding LDR values annotated in each panel.

Responsivity (*R_λ_*) quantifies the photocurrent generated per unit incident light irradiance at a given wavelength *λ*. The *R_λ_* was calculated using:

|  | $R_{\lambda}=\frac{I_{ph}}{I_{irr}\cdot A}$ | (S1) |
| --- | --- | --- |

where *I*_ph_ is the photocurrent, *I*_irr_ is the incident light irradiance, and *A* is the effective active area of the device.

The linear dynamic range (LDR) quantifies the range of incident light irradiance over which the photocurrent varies linearly with irradiance. The LDR was calculated using:

$LDR=20\log_{10}\left(\frac{E_{\max}}{E_{\min}}\right)$ (S2)

where E_max_ and E_min_ are the upper and lower irradiance bounds of this range.^[S18]^

**Figure S9. Specific detectivity of the photodiode.** Specific detectivity (*D*^*^) of the device under 625 nm (VIS) and 850 nm (NIR) illumination measured at −0.5 V and +0.5 V as a function of irradiance (10 nW cm^−2^ to 1 mW cm^−2^).

Detectivity (*D*^*^) represents the minimum detectable signal under noise-limited operation. In this work, *D*^*^ at 0 V was not evaluated; near zero bias, the dark current approaches the instrumental noise floor, causing the shot-noise-limited approximation to underestimate the actual noise contribution and yield artificially inflated, nonphysical detectivity values.^[S19]^ Accordingly, *D*^*^ is reported only at −0.5 and +0.5 V, where the noise assumptions underlying the calculation remain valid. The *D*^*^ was calculated as follows:

|  | $D^{*}=\frac{R_{\lambda}}{\sqrt{2\cdot q\cdot I_{d}\cdot A^{-1}}}$ | (S3) |
| --- | --- | --- |

where *R*_λ_ is the responsivity, *q* is the elementary charge, *I*_d_ is the dark current, and *A* is the effective active area of the device. *D*^*^ was estimated under the shot-noise-limited assumption, in which the detector noise is dominated by dark-current shot noise.^[S19]^

**Figure S10. Rise and decay times of the device under ±0.5 V conditions.**

The rise time was defined as the time required for the photocurrent to reach 90% of its saturation value upon illumination. The decay time corresponds to the time required for the signal to return to 10% of its saturation level.

**Figure S11. Operational repeatability of the photodiode.** Steady-state photocurrent recorded under 850 nm light pulses (2 s off / 3 s on) for 100 cycles (500 s) at biases of (a) −0.5 V, (b) 0 V, and (c) +0.5 V. The left and right inset figures show magnified views of the current response during the first and last 20 s, respectively, highlighting the stable response over repeated cycling.

**Figure S12. Long-term stability of the device.** Photocurrent of the device measured under VIS (625 nm) and NIR (850 nm) illumination over 10 days at applied biases of −0.5 V, 0 V, and +0.5 V. The device was encapsulated using a 0.1-mm-thick glass cover attached with epoxy resin and stored in a nitrogen-filled glovebox.

**Table S1. Comparison of the NL-BPD with previously reported bias-modulated dual-band photodetectors.** Values are listed as band 1 / band 2 in the order given under "Bands". Aside from the present work, all reported photodetectors exhibit linear, quasi-linear, or sublinear irradiance-dependent photoresponses.

| **Device (absorbers)** | **Bands** | **Operating bias (V)** | ***R***  **(A W^−1^)** | ***D** (Jones)** | **LDR (dB)** | **Ref.** |
| --- | --- | --- | --- | --- | --- | --- |
| MAPbI_3_/Si (NL-BPD) | VIS / NIR | −0.5, 0, +0.5 | 0.67 / 1.1 | 6.4×10^11^ / 1.1×10^12^ | ≥100 / ≥100 | This work |
| MAPbI_3_/Si | VIS / NIR | +3 / −3 | 2.5 / 30 | 7.7×10^11^ / 8.9×10^12^ | — | [S20] |
| MAPbI_3_/black Si | VIS / NIR | −1 / +1 | 0.28 / 1.60 | 7.20×10^11^ / 3.19×10^12^ | — | [S21] |
| MAPbI_3-x_Br_x_ / n-MAPbBr_3_ / MAPbI_3_ | VIS / NIR | −3 / +3 | ~0.13 / 0.244 | ~10^12^ / ~10^12^ | ~109 / ~100 | [S22] |
| Perovskite/organic | VIS / NIR^a^ | +0.1 / −0.5 | 0.345 / 0.325 | 3.38×10^13^ / 3.19×10^13^ | 105 / 120 | [S23] |
| Organic/organic (PM type) | VIS / NIR | +15 / −40 | 7.47 / 8.66 | ~10^13^ / ~10^13^ | 92 / 120 | [S24] |
| Organic/organic | VIS / NIR | +1 / −1 | 0.325 / 0.435 | 2.0×10^12^ / 8.8×10^10 b^ | — | [S25] |
| Organic/organic | VIS / NIR–SWIR | +0.5 / −0.6 | 0.2 / 0.1 | 7.8×10^9^ / 1.2×10^9^ ^b^ | 49 / 46^c^ | [S26] |
| Organic/organic | NIR-I / NIR-II | +1 / −1  (and ±3 V) | 0.10 / 0.05 | 6.0×10^11^ / 8.0×10^9^ ^b^ | 85 / 83^d^ | [S27] |
| Ge/Si | VIS–NIR / NIR | +1 / −1 | 0.41 / 0.63 | 7×10^11^ / 2×10^10^ | 124 / 134 | [S28] |
| FAPbBr_3_/MAPbI_3_ | VIS / VIS^e^ | + 0.5 / −1 | 0.33 / 1.5 | 1.8×10^12^ / 8.1×10^12^ | — | [S29] |

“—”: not reported. PM: photomultiplication. ^a^Band separation exhibits some residual spectral overlap: in the NIR mode (−0.5 V), the visible-light responsivity is approximately 15% of the NIR peak responsivity (reported spectral crosstalk below −10 dB). ^b^Calculated from measured noise; all other *D*^*^ values assume shot-noise-limited operation. ^c^Defined as 10 log (*P*_max_/*P*_min_); all other LDR values use 20 log. ^d^Measured at ±3 V. ^e^Two distinct, overlapping visible-light spectral responses.

**Figure S13. Schematic of the control device (Au/HCP/Si, Dev 6) and its corresponding *I*–*V* characteristics.**

**Figure S14. Energy band diagrams and mechanistic analysis of photocurrent generation in the NL-BPD.**

**(Top)** Flat-band energy diagram of the monolithic NP^+^IN hybrid structure, illustrating the energy alignment between N-Si, PEDOT:PSS (p-type), MAPbI_3_ (intrinsic), and PCBM (n-type).

**(Bottom)** Schematic representation of energy band bending and carrier transport mechanisms under positive, zero, and negative bias conditions.

**Photoconductive (PC) mode in Si bulk**: Dominates under applied bias, where external fields drive high photocurrent extraction from the silicon bulk.

**Photovoltaic (PV) mode at junctions**: Governs carrier separation within the depletion regions of the stacked junctions.

**Therefore, the bias-modulated net current** arises from the competition and cooperation between PV-driven separation at the junctions and PC-driven transport in the device, enabling the unique transition from linear to bipolar photoresponses and forming the basis for spectral discrimination.

**Figure S15. Photocurrent traces under simultaneous VIS and NIR illumination.** The photoresponse was measured at different operating biases (−0.5 V, 0 V, and 0.5 V) under simultaneous VIS and NIR pulses. The VIS irradiance was fixed at 100 μW cm^−2^ while the NIR irradiance was varied from 10 to 500 μW cm^−2^.

**Figure S16. Quantitative reconstruction performance of multibias spectral decoding under mixed VIS–NIR illumination.** (a,b) Coefficient of determination (R^2^) and mean absolute error (MAE) obtained using two-bias inputs (−0.5 V, 0 V) for four regression models: Ridge regression, XGBoost, multilayer perceptron (MLP), and Gaussian process (GP). (c,d) Corresponding R^2^ and MAE values obtained using three-bias inputs (−0.5 V, 0 V, +0.5 V).

The R^2^ metric reflects how well the model captures variance in the measured data, with values approaching unity indicating superior predictive accuracy. The MAE metric quantifies the average deviation between predicted and true values, where smaller values correspond to higher reconstruction fidelity.^[S30]^

**Machine Learning Framework and Model Specifications**

To evaluate the sensor’s capability for both light intensity estimation and plant health diagnosis, we employed four distinct machine learning algorithms tailored to each task. Input features comprised current measurements at discrete voltage levels (V = −0.5, 0, 0.5 V), which were standardized using z-score normalization before training. Given the limited dataset size, model generalization was validated using Leave-One-Out Cross-Validation (LOOCV).

For light intensity estimation (regression), we established a Ridge regression model with L2 regularization (=1.0) as a linear baseline. To capture non-linear relationships, we implemented a Multi-layer Perceptron (MLP) with two hidden layers (32 and 16 neurons) utilizing ReLU activation, optimized via the L-BFGS solver (maximum iterations = 2000) for stability on the small dataset. We also employed a Gaussian Process Regressor (GPR) with a Matern kernel (=1.5) combined with a White kernel to explicitly model signal noise. Additionally, an XGBoost ensemble was configured with a maximum depth of 3, a learning rate of 0.03, and 800 estimators to maximize regression accuracy.

**Figure S17. Validation of the physical significance of the bipolar response via permutation testing.** Parity plots showing the reconstruction performance for 625 nm and 850 nm irradiance when the +0.5 V photocurrent input is randomly permuted while preserving the −0.5 V and 0 V inputs. The predictions exhibit substantial scatter and fail to converge to the identity line (dotted line). This control experiment confirms that the high reconstruction fidelity observed in Figure 5c arises specifically from the wavelength-dependent information encoded in the +0.5 V response, rather than a mere increase in input dimensionality.

**Figure S18. Oximetry pulse traces.** a) Pulse waveforms recorded separately under 625 nm and 850 nm illumination at −0.5 V, 0 V, and 0.5 V to validate individual spectral contributions to the mixed signal. b) Stability of the photoresponse under simultaneous 625 and 850 nm illumination, recorded over a 60-second interval at −0.5 V, 0 V, and +0.5 V, demonstrating reliable performance for continuous physiological monitoring. The inset shows zoomed-in pulse traces.

**Figure S19. Experimental setup for NDVI analysis.** Schematic representation of the setup used for NDVI measurements. Reflected light from various leaf samples—categorized as healthy (H), stressed (S), and dead (D)—was projected onto the device's active area, and the resulting photoresponses were recorded under ambient lighting conditions.

**Machine Learning Framework and Model Specifications**

For plant health classification, we compared Logistic Regression (multinomial L-BFGS with L2 regularization) against non-linear classifiers. The Multi-layer Perceptron (MLP) classifier was implemented with the same architecture as the regression model (32 and 16 neurons), optimized via L-BFGS. A Gaussian Process Classifier (GPC) utilizing a Radial Basis Function (RBF) kernel was employed to capture complex decision boundaries. Finally, the XGBoost classifier was configured with reduced complexity (100 estimators, learning rate = 0.1, max depth = 3) to prevent overfitting in the classification task.

**Figure S20. Steady-state photocurrents for NDVI.** Leaf health monitoring under simultaneous and separate (VIS and NIR) illumination. a) Photocurrent recorded at different operating biases for healthy, stressed, and dead leaf samples under simultaneous 625 nm and 850 nm illumination. b) Corresponding photoresponse measured separately under VIS and NIR illumination to validate the individual spectral contributions to the combined signal.

**Supplementary Note 1. Photovoltaic and Photoconductive Effects**

The photovoltaic (PV) effect originates from the generation and separation of photocarriers by the built-in electric field at a semiconductor junction, such as a p–n, p–i–n, or Schottky junction.^[S31]^ When incident photons with energies exceeding the bandgap are absorbed within or near the depletion region, electron–hole pairs are generated. These photogenerated carriers are then separated by the built-in electric field, which drives electrons toward the n-type region and holes toward the p-type region, thereby producing a photocurrent without the need for an external bias.^[S32]^ Under reverse bias, the depletion width and internal electric field increase, which enhances carrier separation and collection efficiency. In contrast, zero bias or forward bias reduces the effective built-in potential, thereby suppressing the photovoltaic photocurrent. Accordingly, PV operation is fundamentally governed by junction electrostatics and field-driven carrier separation within the depletion region.

The photoconductive (PC) mechanism, in contrast, is based on an increase in the electrical conductivity of a semiconductor under illumination.^[S33]^ When photons with energies greater than the bandgap are absorbed, excess electron–hole pairs are generated, increasing the free-carrier concentration and decreasing the material resistivity.^[S34]^ In the absence of a sufficiently strong built-in electric field, an external bias is generally required to drive carrier transport and produce a measurable photocurrent; therefore, at zero bias, the photoconductive contribution is typically negligible. Under an applied electric field, the photogenerated carriers drift toward the respective electrodes, resulting in a measurable photocurrent. The magnitude of this photocurrent is determined by both the carrier generation rate and the carrier lifetime.

Notably, photoconductive gain can occur when the carrier lifetime (τ) exceeds the carrier transit time (τ_tr_). This higher gain effectively amplifies the external photocurrent in proportion to the applied bias, as a stronger electric field reduces the transit time (τ_tr_) and thus enhances the number of circulating charges.

Thus, photoconductive effects are generally more pronounced in the bulk region, where carriers can accumulate and contribute to conductivity modulation. In contrast, within junction regions, photogenerated carriers are rapidly separated and extracted by the built-in electric field before substantial carrier accumulation can occur. Consequently, the distinct voltage dependence of the PC-dominant NIR response, contrasted with the PV-dominant VIS response, enables the bias-tunable spectral discrimination

**Supplementary Note 2. Calculation of Peripheral Oxygen Saturation and Pulse Rate**

Pulse oximetry is a noninvasive monitoring technique that determines pulsatile blood flow and peripheral oxygen saturation (SpO_2_) by leveraging the differential absorption of blood in the red and NIR spectral regions.^[S35]^ Pulse rate and peripheral oxygen saturation are extracted from photoplethysmography (PPG) signals acquired under simultaneous visible (625 nm) and NIR (850 nm) illumination.

When light at these wavelengths propagates through biological tissue, the detected optical signal contains both a DC component, corresponding to static absorption from tissue, venous blood, and bone, and an AC component, originating from pulsatile arterial blood flow synchronized with the cardiac cycle. The AC component is directly associated with arterial hemoglobin and is therefore used for SpO_2_ estimation.^[S36]^ Pulse rate can be determined directly from the time-domain PPG waveforms by calculating the average beat-to-beat interval between consecutive systolic peaks over a stable measurement window:

|  | $\text{Pulse Rate (bpm)}=\frac{60}{\Delta t_{avg}(s)}$ | (S4) |
| --- | --- | --- |

To quantify the oxygenation levels, the ratio-of-ratios (R) is calculated as:

|  | $R=\frac{(AC_{625}/DC_{625})}{(AC_{850}/DC_{850})}$ | (S5) |
| --- | --- | --- |

where AC_λ_ and DC_λ_ represent the pulsatile and baseline components of the detected signal at wavelength λ, respectively. SpO_2_ is then derived from the ratio *R* using the modified Beer–Lambert relation:

|  | $SpO_{2}\approx\frac{\varepsilon_{Hb,625}-R\cdot\varepsilon_{Hb,850}}{(\varepsilon_{Hb,625}-\varepsilon_{HbO_{2},625})+R\cdot(\varepsilon_{HbO_{2},850}-\varepsilon_{Hb,850})}$ | (S6) |
| --- | --- | --- |

where *ε_HbO2,_*_λ_ and *ε_Hb,_*_λ_ are the molar extinction coefficients of HbO_2_ and Hb at wavelength λ. The molar extinction coefficients used for this calculation were obtained from the Prahl hemoglobin absorption database and are summarized in Table S3.^[S37]^ For the 625 nm light source, the coefficients for 626 nm were used as the nearest available data point.

**Supplementary Note 3. Principle of the Normalized Difference Vegetation Index (NDVI)**

The Normalized Difference Vegetation Index (NDVI) is a representative agro-environmental metric that quantifies vegetation vitality by leveraging the reflectance contrast between VIS and NIR light.^[S38]^ NDVI exploits the distinct spectral response of vegetation, where chlorophyll strongly absorbs red light while leaf cellular structures exhibit high reflectance in the NIR region.^[S39]^

NDVI is defined as:

|  | $\text{NDVI=}\frac{R_{NIR}-R_{red}}{R_{NIR}+R_{red}}$ | (S7) |
| --- | --- | --- |

where *R*_NIR_ and *R*_red_ represent the reflected irradiance at NIR and red wavelengths, respectively.^[S40]^ NDVI values range from −1 to +1, with higher positive values indicating dense and healthy vegetation, while values near zero or negative correspond to non-vegetated surfaces. Monitoring NDVI variations enables non-destructive assessment of plant physiological conditions.

**Table S2.** **Simultaneous VIS/NIR illumination database.** A 6 × 6 simultaneous illumination matrix (36 total conditions) was constructed by independently varying the irradiances of 625 nm and 850 nm light sources. Each entry in the database represents the measured photocurrent values under a given pair of irradiances.

| **Photocurrent (μA)**  **@ −0.5 V** | | **850 nm Irradiance (μW cm^−2^)** | | | | | |
| --- | --- | --- | --- | --- | --- | --- | --- |
|  |  | **31.1** | **40.1** | **50** | **62.2** | **73** | **84.1** |
| **625 nm Irradiance (μW cm^−2^)** | **36.2** | −8.4 | −9.7 | −11.2 | −12.9 | −14.5 | −16.1 |
|  | **43** | −9.1 | −10.4 | −11.8 | −13.6 | −15.1 | −16.7 |
|  | **49.9** | −9.7 | −11.0 | −12.5 | −14.2 | −15.7 | −17.3 |
|  | **57.1** | −10.4 | −11.7 | −13.1 | −14.9 | −16.4 | −17.9 |
|  | **64.4** | −11.0 | −12.4 | −13.8 | −15.5 | −17.0 | −18.6 |
|  | **71.8** | −11.7 | −13.1 | −14.5 | −16.2 | −17.7 | −19.2 |

| **Photocurrent (μA)**  **@ 0 V** | | **850 nm Irradiance (μW cm^−2^)** | | | | | |
| --- | --- | --- | --- | --- | --- | --- | --- |
|  |  | **31.1** | **40.1** | **50** | **62.2** | **73** | **84.1** |
| **625 nm Irradiance (μW cm^−2^)** | **36.2** | −3.5 | −3.8 | −4.0 | −4.2 | −4.4 | −4.6 |
|  | **43** | −3.7 | −3.9 | −4.1 | −4.3 | −4.5 | −4.6 |
|  | **49.9** | −3.8 | −4.0 | −4.2 | −4.4 | −4.5 | −4.7 |
|  | **57.1** | −3.9 | −4.1 | −4.3 | −4.5 | −4.6 | −4.7 |
|  | **64.4** | −4.0 | −4.2 | −4.4 | −4.5 | −4.7 | −4.8 |
|  | **71.8** | −4.1 | −4.3 | −4.4 | −4.6 | −4.7 | −4.9 |

| **Photocurrent (nA)**  **@ 0.5 V** | | **850 nm Irradiance (μW cm^−2^)** | | | | | |
| --- | --- | --- | --- | --- | --- | --- | --- |
|  |  | **31.1** | **40.1** | **50** | **62.2** | **73** | **84.1** |
| **625 nm Irradiance (μW cm^−2^)** | **36.2** | 60.8 | 102.7 | 146.7 | 201.7 | 249.6 | 301.4 |
|  | **43** | 61.3 | 102.0 | 146.3 | 201.9 | 250.8 | 302.7 |
|  | **49.9** | 61.3 | 102.7 | 147.1 | 202.9 | 251.5 | 302.2 |
|  | **57.1** | 60.9 | 102.9 | 148.2 | 203.2 | 253.0 | 304.6 |
|  | **64.4** | 63.5 | 104.7 | 148.9 | 205.4 | 253.8 | 306.2 |
|  | **71.8** | 63.5 | 105.2 | 150.4 | 205.7 | 256.0 | 307.5 |

A data-driven decoding algorithm was implemented using the three-bias photocurrent vector (I_−0.5V_, I_0V_, I_+0.5V_) as input variables. The multi-bias responses encode the spectral information embedded in mixed optical signals into distinctive electrical signatures, thereby forming a training database that enables quantitative reconstruction of VIS and NIR components even within a single-pixel configuration.

**Table S3. Molar extinction coefficients used for SpO_2_ calculation.** Values were sourced from the Prahl database.^[S37]^

| **Wavelength (λ)**  **(nm)** | **Molar Extinction Coefficients (ε) (cm^−1^ M^−1^)** | |
| --- | --- | --- |
|  | **HbO_2_** | **Hb** |
| 626 | 707.6 | 5620 |
| 850 | 1058 | 691.32 |

**Table S4.** **Photocurrent measurements for GP model training.** Photocurrent values recorded under simultaneous 625 nm and 850 nm illumination for 30 leaf samples (10 healthy, 10 stressed, and 10 dead) at −0.5 V, 0 V, and +0.5 V bias. Measurements were performed at varying positions across the leaf samples to evaluate signal reproducibility and to construct the training dataset.

|  | **Bias**  **(V)** | **Photocurrent Measurements (μA)** | | | | | | | | | | **Mean (μA)** |
| --- | --- | --- | --- | --- | --- | --- | --- | --- | --- | --- | --- | --- |
|  |  | **1** | **2** | **3** | **4** | **5** | **6** | **7** | **8** | **9** | **10** |  |
| **Healthy Leaves** | **−0.5** | −8.31 | −8.28 | −8.26 | −8.23 | −8.23 | −8.26 | −8.23 | −8.22 | −8.23 | −8.22 | −8.25 |
|  | **0** | −1.51 | −1.50 | −1.50 | −1.50 | −1.50 | −1.50 | −1.50 | −1.50 | −1.50 | −1.50 | −1.50 |
|  | **0.5** | 0.424 | 0.424 | 0.423 | 0.423 | 0.423 | 0.424 | 0.424 | 0.423 | 0.423 | 0.424 | 0.424 |
| **Stressed Leaves** | **−0.5** | −7.74 | −7.75 | −7.74 | −7.75 | −7.74 | −7.74 | −7.74 | −7.73 | −7.73 | −7.73 | −7.74 |
|  | **0** | −1.50 | −1.50 | −1.50 | −1.50 | −1.50 | −1.49 | −1.49 | −1.48 | −1.48 | −1.48 | −1.49 |
|  | **0.5** | 0.380 | 0.380 | 0.381 | 0.382 | 0.382 | 0.383 | 0.384 | 0.384 | 0.385 | 0.384 | 0.382 |
| **Dead Leaves** | **−0.5** | −5.81 | −5.82 | −5.82 | −5.82 | −5.82 | −5.82 | −5.82 | −5.82 | −5.82 | −5.82 | −5.82 |
|  | **0** | −1.33 | −1.33 | −1.33 | −1.33 | −1.33 | −1.32 | −1.32 | −1.32 | −1.32 | −1.32 | −1.32 |
|  | **0.5** | 0.258 | 0.260 | 0.260 | 0.261 | 0.261 | 0.261 | 0.261 | 0.262 | 0.262 | 0.262 | 0.261 |

**Table S5. Power meter measurements for reference NDVI determination and state classification.** The reflected intensities of red and NIR light were measured at varying positions across 30 leaf samples (10 healthy, 10 stressed, and 10 dead) using a calibrated power meter. The resulting mean reflected intensities were utilized to calculate the reference NDVI for each health state, thereby providing a quantitative basis for defining the three physiological health states (healthy, stressed, and dead).

|  | **λ**  **(nm)** | **Irradiance Measurements (μW cm^−2^)** | | | | | | | | | | **Mean** | **NDVI** |
| --- | --- | --- | --- | --- | --- | --- | --- | --- | --- | --- | --- | --- | --- |
|  |  | **1** | **2** | **3** | **4** | **5** | **6** | **7** | **8** | **9** | **10** |  |  |
| **Healthy Leaves** | **625** | 3.2 | 4.2 | 5.9 | 3.6 | 4.5 | 4.0 | 4.2 | 4.3 | 3.2 | 3.4 | 4.05 | 0.71 |
|  | **850** | 19.5 | 24 | 27.8 | 22.1 | 26.7 | 23.4 | 25.3 | 24.5 | 20 | 24.6 | 23.79 |  |
| **Stressed Leaves** | **625** | 3.18 | 3.4 | 4.3 | 4.7 | 3.8 | 4.2 | 3.75 | 2.5 | 3.3 | 4.9 | 3.8 | 0.63 |
|  | **850** | 14.3 | 15.93 | 18.5 | 20 | 17.6 | 19.1 | 16.8 | 12.6 | 15.5 | 19 | 16.93 |  |
| **Dead Leaves** | **625** | 7.24 | 8.3 | 7.8 | 7.07 | 6.6 | 7.22 | 7.36 | 7.9 | 6.82 | 6.79 | 7.31 | 0.42 |
|  | **850** | 17.1 | 19.1 | 19.4 | 16.8 | 16.4 | 18.1 | 18.4 | 18.7 | 17.2 | 16.2 | 17.74 |  |

**References**

[S1] O. Yossef Hay, M. Cohen, I. Nitzan, Y. Kasirer, S. Shahroor-Karni, Y. Yitzhaky, S. Engelberg, M. Nitzan, Pulse oximetry with two infrared wavelengths without calibration in extracted arterial blood. Sensors **18**, 3457 (2018). <https://doi.org/10.3390/s18103457>
[S2] C.M. Lochner, Y. Khan, A. Pierre, A.C. Arias, All-organic optoelectronic sensor for pulse oximetry. Nat. Commun. **5**, 5745 (2014). <https://doi.org/10.1038/ncomms6745>
[S3] M. Wald, P. Erwin, N. Annon-Eberharter, T. Werther, LED light can falsify pulse oximetry readings via the stroboscopic effect. Phys. Eng. Sci. Med. **46**, 1667 (2023). <https://doi.org/10.1007/s13246-023-01328-2>
[S4] R. Muhammad, K.T. Htun, E.E. Nettey-Oppong, A. Ali, D.K. Jeon, H.-W. Jeong, K.M. Byun, S.H. Choi, Pulse oximetry imaging system using spatially uniform dual wavelength illumination. Sensors **23**, 3723 (2023). <https://doi.org/10.3390/s23073723>
[S5] J.D. Stamford, S. Vialet-Chabrand, I. Cameron, T. Lawson, Development of an accurate low cost NDVI imaging system for assessing plant health. Plant Methods **19**, 9 (2023). <https://doi.org/10.1186/s13007-023-00981-8>
[S6] Q. Zhao, Y. Qu, The retrieval of ground NDVI (normalized difference vegetation index) data consistent with remote-sensing observations. Remote Sens. **16**, 1212 (2024). <https://doi.org/10.3390/rs16071212>
[S7] P. Fan, D. Gu, G.-X. Liang, J.-T. Luo, J.-L. Chen, Z.-H. Zheng, D.-P. Zhang, High-performance perovskite CH3NH3PbI3 thin films for solar cells prepared by single-source physical vapour deposition. Sci. Rep. **6**, 29910 (2016). <https://doi.org/10.1038/srep29910>
[S8] M. Reiser, A. Breidenassel, O. Amft, Impact of sensor configuration and melanin concentration on reflective pulse oximetry using Monte Carlo simulations. Sci. Rep. **15**, 39363 (2025). <https://doi.org/10.1038/s41598-025-26560-6>
[S9] Q. Liu, A comparative study of different normalized difference vegetation indices from the wide band spectral imager of Tiangong II, China. Proc. SPIE **10806**, 1080637 (2018). <https://doi.org/10.1117/12.2503089>
[S10] J. Cameron, P.J. Skabara, The damaging effects of the acidity in PEDOT:PSS on semiconductor device performance and solutions based on non-acidic alternatives. Mater. Horiz. **7**, 1759 (2020). <https://doi.org/10.1039/C9MH01978B>
[S11] Y.-F. Chen, Y.-T. Tsai, L. Hirsch, D.M. Bassani, Kinetic isotope effects provide experimental evidence for proton tunneling in methylammonium lead triiodide perovskites. J. Am. Chem. Soc. **139**, 16359 (2017). <https://doi.org/10.1021/jacs.7b09526>
[S12] Q.B. Ke, J.-R. Wu, C.-C. Lin, S.H. Chang, Understanding the PEDOT:PSS, PTAA and P3CT-X hole-transport-layer-based inverted perovskite solar cells. Polymers **14**, 823 (2022). <https://doi.org/10.3390/polym14040823>
[S13] F.M. Rombach, A. Dasgupta, M. Kober-Czerny, H. Jin, J.M. Ball, J.A. Smith, M.D. Farrar, H.J. Snaith, Disentangling degradation pathways of narrow bandgap lead-tin perovskite material and photovoltaic devices. Nat. Commun. **16**, 5450 (2025). <https://doi.org/10.1038/s41467-025-58489-9>
[S14] M. Yi, W. Jang, D.H. Wang, Controlled pH of PEDOT:PSS for reproducible efficiency in inverted perovskite solar cells: Independent of active area and humidity. ACS Sustainable Chem. Eng. **7**, 8245 (2019). <https://doi.org/10.1021/acssuschemeng.8b06619>
[S15] X. Fan, N.E. Stott, J. Zeng, Y. Li, J. Ouyang, L. Chu, W. Song, PEDOT:PSS materials for optoelectronics, thermoelectrics, and flexible and stretchable electronics. J. Mater. Chem. A **11**, 18561 (2023). <https://doi.org/10.1039/D3TA03213B>
[S16] S. Jäckle, M. Mattiza, M. Liebhaber, G. Brönstrup, M. Rommel, K. Lips, S. Christiansen, Junction formation and current transport mechanisms in hybrid n-Si/PEDOT:PSS solar cells. Sci. Rep. **5**, 13008 (2015). <https://doi.org/10.1038/srep13008>
[S17] Z.T. Gebremichael, C. Ugokwe, S. Alam, S. Stumpf, M. Diegel, U.S. Schubert, H. Hoppe, How varying surface wettability of different PEDOT:PSS formulations and their mixtures affects perovskite crystallization and the efficiency of inverted perovskite solar cells. RSC Adv. **12**, 25593 (2022). <https://doi.org/10.1039/D2RA03843A>
[S18] C. Li, H. Wang, F. Wang, T. Li, M. Xu, H. Wang, Z. Wang, X. Zhan, W. Hu, L. Shen, Ultrafast and broadband photodetectors based on a perovskite/organic bulk heterojunction for large-dynamic-range imaging. Light Sci. Appl. **9**, 31 (2020). <https://doi.org/10.1038/s41377-020-0264-5>
[S19] X. Gong, M. Tong, Y. Xia, W. Cai, J.S. Moon, Y. Cao, G. Yu, C.-L. Shieh, B. Nilsson, A.J. Heeger, High-detectivity polymer photodetectors with spectral response from 300 nm to 1450 nm. Science **325**, 1665 (2009). <https://doi.org/10.1126/science.1176706>
[S20] W. Kim, Y. Seo, D. Ahn, I.S. Kim, C. Balamurugan, G.Y. Jung, S. Kwon, H. Kim, Y. Pak, Monolithic perovskite–silicon dual-band photodetector for efficient spectral light discrimination. Adv. Sci. **11**, 2308840 (2024). <https://doi.org/10.1002/advs.202308840>
[S21] J. Sung, M.S. Kim, H. Lee, G.J. Lee, H. Kim, S. Kwon, Low-bias, broadband photodetection enabled by MAPbI3/black silicon dual-mode photodetector for compact optoelectronic platforms. Chem. Eng. J. **527**, 171589 (2026). <https://doi.org/10.1016/j.cej.2025.171589>
[S22] J. Zhao, X. Wang, Y. Xu, Y. Pan, Y. Li, J. Chen, Q. Li, X. Zhang, Z. Zhu, Z. Zhao, E.E. Elemike, D.C. Onwudiwe, B.S. Bae, S.B. Shafie, W. Lei, Electrically modulated near-infrared/visible light dual-mode perovskite photodetectors. ACS Appl. Mater. Interfaces **14**, 25824 (2022). <https://doi.org/10.1021/acsami.2c01796>
[S23] X. Xu, Q. Liu, C. Shan, R. Li, X. Xu, T. Dai, Y. Bao, S. Zhou, D.H. Wang, A.K.K. Kyaw, Synergistic charge generation and collection enabled by incorporation of crystalline polymer for ultrafast-response dual-band perovskite/organic photodetectors. ACS Appl. Mater. Interfaces **17**, 62388 (2025). <https://doi.org/10.1021/acsami.5c17019>
[S24] Z. Lan, Y. Lei, W.K.E. Chan, S. Chen, D. Luo, F. Zhu, Near-infrared and visible light dual-mode organic photodetectors. Sci. Adv. **6**, eaaw8065 (2020). <https://doi.org/10.1126/sciadv.aaw8065>
[S25] Z. Lan, Y.S. Lau, L. Cai, J. Han, C.W. Suen, F. Zhu, Dual-band organic photodetectors for dual-channel optical communications. Laser Photonics Rev. **16**, 2100602 (2022). <https://doi.org/10.1002/lpor.202100602>
[S26] N. Li, N. Eedugurala, J.D. Azoulay, T.N. Ng, A filterless organic photodetector electrically switchable between visible and infrared detection. Cell Rep. Phys. Sci. **3**, 100711 (2022). <https://doi.org/10.1016/j.xcrp.2021.100711>
[S27] Y. Tang, Z. Wang, M. Gao, J. Han, L. Yuan, F. Zhu, Dual-mode narrowband organic photodetectors for self-aligned imaging in NIR-I and NIR-II. Nat. Commun. **16**, 7144 (2025). <https://doi.org/10.1038/s41467-025-62394-6>
[S28] E.T. Simola, A. De Iacovo, J. Frigerio, A. Ballabio, A. Fabbri, G. Isella, L. Colace, Voltage-tunable dual-band Ge/Si photodetector operating in VIS and NIR spectral range. Opt. Express **27**, 8529 (2019). <https://doi.org/10.1364/OE.27.008529>
[S29] H. Kim, W. Kim, Y. Pak, T.J. Yoo, H.W. Lee, B.H. Lee, S. Kwon, G.Y. Jung, Bias-modulated multicolor discrimination enabled by an organic–inorganic hybrid perovskite photodetector with a p-i-n-i-p configuration. Laser Photonics Rev. **14**, 2000305 (2020). <https://doi.org/10.1002/lpor.202000305>
[S30] A. Jierula, S. Wang, T.-M. Oh, P. Wang, Study on accuracy metrics for evaluating the predictions of damage locations in deep piles using artificial neural networks with acoustic emission data. Appl. Sci. **11**, 2314 (2021). <https://doi.org/10.3390/app11052314>
[S31] A.S. Al-Ezzi, M.N.M. Ansari, Photovoltaic solar cells: A review, Appl. Syst. Innov. **5**, 67 (2022). <https://doi.org/10.3390/asi5040067>
[S32] S.M. Sze, Y. Li, K.K. Ng, Physics of Semiconductor Devices, 4th edn. (John Wiley & Sons, Hoboken, NJ, 2021), Chap. 13.
[S33] G.E. Stillman, Optoelectronics, In: W.M. Middleton, M.E. Van Valkenburg (Eds.), Reference Data for Engineers: Radio, Electronics, Computer, and Communications, 9th edn. (Newnes, Woburn, MA, 2002), pp. 21-1–21-31. <https://doi.org/10.1016/B978-075067291-7/50023-6>
[S34] B.G. Streetman, S.K. Banerjee, Solid State Electronic Devices, 7th edn. (Pearson, Upper Saddle River, NJ, 2015), Chap. 4.
[S35] E.D. Chan, M.M. Chan, M.M. Chan, Pulse oximetry: Understanding its basic principles facilitates appreciation of its limitations. Respir. Med. **107**, 789 (2013). <https://doi.org/10.1016/j.rmed.2013.02.004>
[S36] Y. Khan, D. Han, A. Pierre, J. Ting, X. Wang, C.M. Lochner, G. Bovo, N. Yaacobi-Gross, C. Newsome, R. Wilson, A.C. Arias, A flexible organic reflectance oximeter array. Proc. Natl. Acad. Sci. U. S. A. **115**, E11015 (2018). <https://doi.org/10.1073/pnas.1813053115>
[S37] S. Prahl, Tabulated molar extinction coefficient for hemoglobin in water. Oregon Medical Laser Center. <https://omlc.org/spectra/hemoglobin/summary.html>. Accessed 22 July 2026.
[S38] N. Pettorelli, J.O. Vik, A. Mysterud, J.-M. Gaillard, C.J. Tucker, N.C. Stenseth, Using the satellite-derived NDVI to assess ecological responses to environmental change. Trends Ecol. Evol. **20**, 503 (2005). <https://doi.org/10.1016/j.tree.2005.05.011>
[S39] D.A. Sims, J.A. Gamon, Relationships between leaf pigment content and spectral reflectance across a wide range of species, leaf structures and developmental stages. Remote Sens. Environ. **81**, 337 (2002). <https://doi.org/10.1016/S0034-4257(02)00010-X>
[S40] C.J. Tucker, Red and photographic infrared linear combinations for monitoring vegetation. Remote Sens. Environ. **8**, 127 (1979). <https://doi.org/10.1016/0034-4257(79)90013-0>
